# Computational Modeling of NLRP3 Identifies Enhanced ATP Binding and Multimerization in Cryopyrin-Associated Periodic Syndromes

**DOI:** 10.3389/fimmu.2020.584364

**Published:** 2020-11-19

**Authors:** Jenny Mae Samson, Dinoop Ravindran Menon, Prasanna K. Vaddi, Nazanin Kalani Williams, Joanne Domenico, Zili Zhai, Donald S. Backos, Mayumi Fujita

**Affiliations:** ^1^ Department of Dermatology, University of Colorado Anschutz Medical Campus, Aurora, CO, United States; ^2^ Department of Pharmaceutical Sciences, Skaggs School of Pharmacy, University of Colorado Anschutz Medical Campus, Aurora, CO, United States; ^3^ Department of Immunology, University of Colorado Anschutz Medical Campus, Aurora, CO, United States; ^4^ Denver VA Medical Center, Aurora, CO, United States

**Keywords:** NLRP3, cryopyrin-associated periodic syndrome, NLRP3-AID, familial cold autoinflammatory syndrome, Muckle-Wells Syndrome, chronic infantile neurologic cutaneous and articular syndrome, neonatal-onset multisystem inflammatory disease

## Abstract

Cyropyrin-associated periodic syndromes (CAPS) are clinically distinct syndromes that encompass a phenotypic spectrum yet are caused by alterations in the same gene, *NLRP3.* Many CAPS cases and other *NLRP3*-autoinflammatory diseases (*NLRP3*-AIDs) are directly attributed to protein-coding alterations in *NLRP3* and the subsequent dysregulation of the NLRP3 inflammasome leading to IL-1β-mediated inflammatory states. Here, we used bioinformatics tools, computational modeling, and computational assessments to explore the proteomic consequences of *NLRP3* mutations, which potentially drive NLRP3 inflammasome dysregulation. We analyzed 177 mutations derived from familial cold autoinflammatory syndrome (FCAS), Muckle-Wells Syndrome (MWS), and the non-hereditary chronic infantile neurologic cutaneous and articular syndrome, also known as neonatal-onset multisystem inflammatory disease (CINCA/NOMID), as well as other *NLRP3*-AIDs. We found an inverse relationship between clinical severity and the severity of predicted structure changes resulting from mutations in *NLRP3*. Bioinformatics tools and computational modeling revealed that NLRP3 mutations that are predicted to be structurally severely-disruptive localize around the ATP binding pocket and that specific proteo-structural changes to the ATP binding pocket lead to enhanced ATP binding affinity by altering hydrogen-bond and charge interactions. Furthermore, we demonstrated that NLRP3 mutations that are predicted to be structurally mildly- or moderately-disruptive affect protein-protein interactions, such as NLRP3-ASC binding and NLRP3-NLRP3 multimerization, enhancing inflammasome formation and complex stability. Taken together, we provide evidence that proteo-structural mechanisms can explain multiple mechanisms of inflammasome activation in *NLRP3-*AID.

## Introduction

Inflammasomopathies, types of autoinflammatory diseases, are driven by activation of inflammasomes, leading to IL-1β-mediated conditions with distinct clinical presentations (1–5). Inflammasomes are multiprotein complexes that are assembled in response to various stimuli and responsible for caspase-1-dependent IL-1β activation (6–9). NLRP3 is an inflammasome sensor, whose activation leads to a variety of autoinflammatory diseases (*NLRP3-*AIDs), including three well-documented periodic fever syndromes: familial cold autoinflammatory syndrome (FCAS), Muckle-Wells syndrome (MWS), and chronic infantile neurologic cutaneous and articular syndrome, also known as neonatal-onset multisystem inflammatory disease (CINCA/NOMID) (10–15). The phenotypic spectrum encompassing these three *NLRP3-*associated syndromes is collectively known as cyropyrin-associated periodic syndrome (CAPS) (16–19). Among CAPS, FCAS is the mildest phenotype, requiring a trigger such as cold temperature to cause symptom onset, while CINCA/NOMID is the most severe phenotype with onset in neonates, without a need for a trigger and often with neurological consequences (20). While both FCAS and MWS are known heritable conditions with a familial autosomal dominant pattern of inheritance (21, 22), CINCA/NOMID is sporadic and attributed to *de novo* mutations in *NLRP3*.

NLRP3 is expressed in an autoinhibited form (23, 24). Only upon stimulation initiated through ATP priming (25), does NLRP3 undergo a conformational change, facilitating the homotypic PYR-PYR domain interactions with ASC (6, 26, 27). Bound together, NLRP3-ASC complexes multimerize and recruit procaspase-1 through homotypic CARD-CARD interactions, which bring procaspase-1 into a conformation that facilitates self-cleavage and activation of its catalytic subunits (28, 29). Ultimately, the activation of the effector protein caspase-1 allows for the swift processing of pro-IL1-β into its active form (9, 28, 30–32). Given its complex regulation and downstream effectors, it is understandable that dysregulated NLRP3 leads to the development of AID such as CAPS. While some studies have shown the direct effects of *NLRP3* mutations on increased cytokine production and inflammasome activity in CAPS cases, fewer have investigated the mechanisms of how mutated *NLRP3* drives these human diseases (33, 34).

In this study, we performed a systematic review of publications and databases describing *NLRP3-*AIDs, summarized the clinical and molecular effects of germline *NLRP3* mutations, and explored the structural differences that underlie CAPS mutations. The computational modeling of NLRP3 predicted enhanced ATP binding and multimerization as mechanisms of NLRP3 activation in cryopyrin-associated periodic syndromes.

## Materials and Methods

### Literature Review of *NLRP3-*AID Mutations

A literature review of *NLRP3-*AID mutations was performed in PubMed and Google Scholar by querying “*NLRP3* mutation” and terms including CAPS, AID, FCAS, MWS, CINCA, NOMID, JIA, RA, and disease. Review papers and databases such as Infevers and Online Mendelian Inheritance in Man (OMIM) were also queried for *NLRP3-*AID mutations. Information on clinical phenotypes and molecular consequences from disease reports, case studies, and other primary papers were compiled.

### Criteria for Classification

FCAS: intermittent fever, cold-induced urticarial papules and plaques lasting minutes to several days with associated arthralgias, conjunctivitis and headaches

MWS: intermittent fever, widespread urticarial papules and plaques lasting 1–2 days with associated abdominal pain, myalgias, arthralgias, conjunctivitis, episcleritis, and sensorineural hearing loss

CINCA/NOMID: intermittent fever, widespread, continuous urticarial papules and plaques with associated arthritis, deforming arthropathy, conjunctivitis, uveitis, blindness, sensorineural hearing loss, aseptic meningitis, seizures often presenting at birth

### Structural Bioinformatics Scoring Tools

PolyPhen-2 (35) was used through http://genetics.bwh.harvard.edu/pph2/bgi.shtml, and standard instructions for Batch Query were followed. SIFT (36) and PROVEAN (37, 38) were simultaneously used through http://provean.jcvi.org/protein_batch_submit.php?species=human, and standard instructions for the query were followed. All algorithm inputs utilized the UniProt protein identifier for NLRP3: Q96P20 in addition to the NLRP3 protein FASTA sequence. All results were downloaded in TSV file format or Excel-compatible text format for further analysis.

### Combination Scoring System

To obtain a reliable scoring metric for mutation analysis, we generated a combined score from all three bioinformatics algorithms, PolyPhen-2, SIFT, and PROVEAN. To factor out the unknown distribution of these scores, we ranked them from most to least structurally disruptive. For scores that were tied, the highest rank was applied to each tied score.

Next, we combined the scores in a mathematically meaningful way. This can be done by deriving p-values from rank scores since there are well-defined rules for combining p-values. Under the null hypothesis, the ranks are random, so if the maximum rank is *r_max_*, then the likelihood (probability), *p*, of a given rank, *r*, is as good or better than it was measured to be is:

$$p=\frac{r}{r_{max}}.$$

We used this formula to derive p-values against the null hypothesis for each of the ranks from all three algorithms. Next, we made the simplifying assumptions that 1) the PolyPhen-2 p-values (*p_PolyPhen-2_*) are independent of those from SIFT and PROVEAN (*p_SIFT_*and *p_PROVEAN_*, respectively) since PolyPhen-2 uses a completely different algorithm, 2) *p_SIFT_* and *p_PROVEAN_* behave as dependent variables because PROVEAN is a slightly modified version of the SIFT algorithm, and 3) *p_SIFT_* and *p_PROVEAN_* should be equally weighted since we have no evidence that one is more reliable than the other. Assumption 1 implies that the joint p-value combining all three algorithms, which we term *p_weighted_*, is the product of *p_PolyPhen-2_* and the combined p-values of SIFT and PROVEAN. Assumptions 2 and 3 imply that the *p_SIFT_* and *p_PROVEAN_* should be combined by a multiplicative average. The final equation for this joint probability is:

$$P_{weighted}=p_{PolyPhen2}({\left[p_{SIFT}\times p_{PROVEAN}\right]}^{1/2}).$$

Since we did not know the extent to which assumptions 1–3 would hold, we interpreted *p_weighted_* as a score rather than a probability. However, we considered that the assumptions were sufficiently correct that *p_weighted_* is close to the actual joint p-value against the null hypothesis; therefore, we took *p_weighted_* < 0.05 as a significance threshold.

We tested our combined score by scoring rare NLRP3 single-nucleotide variants (minor allele frequency < 0.001) from gnomAD (39) and compared the averages and score distributions of these to our collection of 177 pathogenic NLRP3-AID variants (**Supplementary Figure 4**).

### Statistical Analysis

Differences were analyzed using unpaired Student’s *t*-test using GraphPad Prism version 8.3.0 (GraphPad Software, San Diego, CA).

### NLRP3 Homology Model

As no structural data currently exists for the human NLRP family or any of its orthologs, we generated several structural homology models of human NLRP3 using the MODELLER protocol (40) within the Discovery Studio 2018 suite (Biovia, Inc, San Diego, CA) and by submitting the protein sequence to the ModWeb and I-Tasser (41) protein modeling servers. The resulting top-scoring models were then subjected to explicit solvent-based molecular dynamics (MD) simulations with YASARA v19.4 (YASARA Biosciences, GmbH, Vienna, Austria) utilizing the YASARA2 force field (42–45), which combines the AMBER (ff14SB) force field (46) with self-parameterizing knowledge-based potentials (47), to refine each model as described previously (43). Refinement simulations were run for one nanosecond (ns) with snapshots taken every 25 picoseconds (ps) and assessed using the WHAT_IF and WHAT_CHECK (48, 49) structure validation tools, which compare model characteristics (dihedral angles, residue packing, etc.) to the average values of ~30,000 high-resolution structures in the PDB (www.rcsb.org) (50). The best-refined model was the model that was generated using the ModWeb server with a quality score of -0.69, indicating that the overall characteristics of the NLRP3 homology model are within one standard deviation of the average values for high-resolution protein crystal structures.

The NLRP3 trimer structure was generated by superimposition and molecular overlay of the NLRP3 monomer homology model onto three consecutive monomers of NLRC4 from the cryo-EM structure of the NAIP2-NLRC4 inflammasome complex (PDB ID: 3JBL) using Discovery Studio 2018 (Biovia, Inc, San Diego, CA).

### NLRP3 Structural Analysis

Structural analysis was performed using the human NLRP3 PYR crystal structure (3QF2) by Bae and Park (51) and the NLRP3 homology model generated as described above. To elucidate the potential functional ramifications of NLRP3 mutations, we made each mutation individually to the WT structure and subjected the WT and each mutant to explicit solvent-based energy minimization with the AMBER (ff14SB) force field (46) to assess predicted alterations in protein structure and surface characteristics. The predicted effects of the mutations on ATP binding were assessed by docking ADP and ATP into the refined WT and mutant structures using the flexible docking protocol (52) in Discovery Studio 2018 (Biovia, Inc, San Diego, CA), and the binding energies were calculated using the AutoDock VINA (53) module within YASARA v19.4 (YASARA Biosciences, GmbH, Vienna, Austria).

To assess the effects of the PYR mutations, we first performed the MD-based refinements of the WT NMR structures of full-length ASC (PDB ID: 2KN6) (54) and the PYR of NLRP3 (PDB ID: 2NAQ) (55) as described above for the homology modeling of NLRP3. The ZDOCK (56), ZRANK (57), and RDOCK (58) algorithms were employed to predict the binding mode of the NLRP3 PYR at each of the two established interaction interfaces of the PYR of ASC in the assembled inflammasome. We modeled the effects of the NLRP3 mutations located at each of these interfaces, as well as those located at the interface of the middle monomer in the assembled NLRP3 trimer structure, using explicit solvent-based energy minimization as described above.

The predicted binding orientations and intermolecular contacts of ATP, as well as the protein-protein interactions in each NLRP structure, were visualized in 2D and 3D using Discovery Studio 2018 (Biovia, Inc., San Diego, CA).

## Results

### *NLRP3-*AID Mutations Are Localized Diversely Within the Coding Sequence

We retrieved reports of *NLRP3-*AIDs and compiled a comprehensive list of germline and somatic mosaic mutations in *NLRP3* in **Table 1**. The mutations are listed by disease phenotype and detail the genetic changes reported for each proteomic change alongside references. In many reports of germline *NLRP3* mutations, the amino acid positions are mismatched to the canonical amino acid positions reported in the US National Library of Medicine NCBI and Ensembl as a result of the protein sequence being counted from the second methionine (M3) instead of M1 due to better alignment of the Kozak consensus sequence with the M3 (59, 128). All mutations in our study have been updated to match the NCBI NLRP3 canonical protein sequence and are counted from M1.

**Table 1:** Literature Review of NLRP3 Germline Mutations.

Disease Phenotype	Proteomic Change (Amino Acid Change) ^[References]^
Familial Cold Autoinflammatory Syndrome (FCAS)	V200M (c.562G>A) (17, 59–63), D213N (c.631G>A) (64), C261W (c.777T>G) (62, 64, 65), R262W (c.778C>T) (17, 60, 64, 66)^; FCAS/MWS overlap syndrome^ (59), V264G (c.785T>G) (64, 67), G303D (c.902G>A) (64, 68), L307P (c.914T>C) (59, 64, 69), L355P (c.1058T>C) (62, 64, 69–70, 71), K377E (c.1123A>G) (64, 72), T438A (c.1306A>G) (64, 73), T438I (c.1307C>T) (19), A441T (c.1315G>A) (69), A441V (c.1316C>T) (11, 64, 69, 70, 74), R490K (c.1463G>A) (64, 75), F525C (c.1568T>G) (76), E527K (c.1573G>A) (64, 77), Y565N (c.1687T>A) (62, 64), E607V (c.1814A>T) (64, 78)^+^, E629G (c.1880A>G) (11, 60, 64, 69, 79), M661K (c.1976T>A) (64, 77)
Muckle-Wells Syndrome (MWS)	D31V (c.86A>T) (64, 80, 81), V72M (c.208G>A) (64, 80), R137H (c.404G>A) (64, 82), R172S (c.508C>A) (64, 83), V200M (c.562G>A) (11, 59–62, 64), R262L (c.779G>T) (64, 69), R262W (c.778C>T) (17, 60, 64, 66)^; FCAS/MWS overlap syndrome^ (59), V264G (c.785T>G) (64, 67), L266V (c.790C>G) (64), T268P (c.796A>C) ^MWS/CINCA overlap syndrome^ (64), D305N (c.907G>A; c.913G>A) (17, 64, 66), E313K (c.931G>A) (10, 64, 70, 84), H314P (c.935A>C) (64, 85), R327W (c.973C>T) (64, 86), T350M (c.1043C>T) (17, 64, 70, 80, 87), A354V (c.1055C>T) (10, 11, 60, 64, 87), W416L (c.1241G>T) (64), A441T (c.1315G>A) (17, 64), Y443H (c.1321T>C) ^MWS/CINCA overlap syndrome^ (64), I482F (c.1438A>T) (64, 88), R490K (c.1463G>A) (70, 75),A497V (c.1484C>T) (64, 75), M523T (c.1562T>C) (64), F525C (c.1568T>G) (62, 64, 89), E527K (c.1573G>A) (34), F568Y (c.1697T>A; c.1703T>A) (64), E569A (c.1700A>C) (64, 83), K570N (c.1704G>C; c.1710G>C) (90), G571R (c.1705G>C) (17, 60, 64, 65, 69), F581Y (c.1736T>A) (64, 91), I600F (c.1792A>T) (64), R605G (c.1807A>G) (64), P651S (c.1945C>T) (64), M703T (c.2102T>C) (64), Q705K (c.2107C>A) (80), S712C (c.2129C>G) (64)
Mosaic MWS	R262P (c.779G>C) (92), D305A (c.908A>C) (64, 83), I336V (c.1000A>G) (83), K357N (c.1065A>T) (83), K357T (c.1064A>C) (64, 83), L413V (c.1231C>G) (64, 83), F525L (c.1569C>A; c.1569C>G) (83), E569K (c.1699G>A) (64, 93), Q638E (c.1906C>G) (64, 94)
Chronic Infantile Neurologic, Cutaneous, and articular syndrome (CINCA/NOMID)	C150Y (c.433G>A) (19, 64), R170Q (c.503G>A) (64), I174T (c.515T>C) (64, 95), K175E (c.517A>G) (61, 64), R262P (c.779G>C) (61, 64, 69), R262Q (c.785G>A; c.779G>A) (64, 96) ^+^, R262W (c.778C>T) (93), V264A (c.785T>C) (62, 64, 76), L266F (c.790C>T) (62, 64, 69–70), L266H (c.791T>A) (64, 66, 89), L266R (c.791T>G) (64), T268P (c.796A>C) (64), D305G (c.908A>G) (64, 69), D305H (c.907G>C) (64, 68), D305N (c.907G>A; c.913G>A) (16, 17, 34, 64, 66, 76, 90–97, 93), E306K (c.910G>A) (64, 98), Q308E (c.916C>G) (64), Q308K (c.916C>A) (64, 69, 97), G309V (c.920G>T) (64, 99), F311S (c.926T>C; c.932T>C) (60, 64, 66, 69, 97), F311Y (c.926T>A) (64), P317L (c.944C>T) (64, 100), G328E (c.977G>A) (64, 101), S333R (c.993C>A) (64, 102), I336V (c.1000A>G) (64), T350M (c.1043C>T) (34, 69), V353L (c.1051G>C; c.1051G>T) (64, 90), V353M (c.1051G>A) (64), A354T (c.1054G>A) (64), E356D (c.1062G>T) (64, 69), H360R (c.1073A>G) (64, 87, 97), A376D (c.1121C>A) (64, 89), A376N (c.1121C>A) (69, 89), T407P (c.1213A>C) (61, 64, 69), M408I (c.1218G>C) (34, 64, 98), M408T (c.1217T>C) (64), T435A (c.1297A>T) (61, 103), T438I (c.1307C>T) (64, 69), T438N (c.1307C>A) (64, 69, 97), T438P (c.1306A>C) (64), A441P (c.1315G>C) (64), A441V (c.1316C>T) (61, 93), Y443H (c.1321T>C) ^MWS/CINCA overlap syndrome^ (64), F445L (c.1329C>G) (62, 64), N479K (c.1431C>A) (34, 64, 93, 98), I482F (c.1438A>T) (64, 88), R490K (c.1463G>A) (75), F525L (c.1569C>A; c.1569C>G) (64, 69, 89), F525Y (c.1568T>A) (64, 104), E527V (c.1574A>T) (64), F568Y (c.1697T>A; c.1703T>A) (90), G571A (c.1706G>C) (64), Y572C (c.1709A>G) (11, 64, 69, 87, 89), Y572F (c.1709A>T) (64), Y572H (c.1708T>C) (64, 105), L573F (c.1713G>T; c.1713G>C) (64), I574F (c.1714A>T) (64), F575S (c.1718T>C) (60, 64, 69, 97), T589I (c.1760C>T) (64), S597G (c.1783A>G; c.1789A>G) (64, 90, 106), I600F (c.1792A>T) (64), R605G (c.1807A>G) (33), E629D (c.1881A>T) (64), L634F (c.1896G>T) (64, 69), M664T (c.1985T>C) (64, 69, 93, 97), E690K (c.2062G>A) (64, 98), E692K (c.2068G>A) (64), S728G (c.2176A>G) (64), G757A (c.2264G>C) (15, 64), G757R (c.2263G>A) (15, 17–19, 64, 93), R779C (c.2329C>T; c.2335C>T) (90), G811S (c.2419G>A) (64, 107), Y861C (c.2576A>G) (13, 64)
Mosaic CINCA/NOMID	S198N (c.587G>A) (14, 64), L266F (c.790C>T) (93, 103),G303S (c.901G>A) (103), F304L (c.906C>A) (108), D305H (c.907G>C) (75, 103, 108, 109), G309D (c.920G>A) (64, 105), G309S (c.919G>A) (64, 93), K357N (c.1065A>T) (64, 103), M408V (c.1216A>G) (64, 103), T435I (c.1298C>T) (64, 92, 103), A441P (c.1315G>C) (103), Y565C (c.1688A>G) (64, 92), G566S (c.1690G>A) (64, 92), F568L (c.1698C>A) (64, 103), E569K (c.1699G>A) (64, 93), K570N (c.1704G>C; c.1710G>C) (64, 103), Y572C (c.1709A>G) (14, 93, 103), G757R (c.2263G>A) (103)
Mosaic *NLRP3-*AID	L266P (c.791T>C) ^Mosaic CAPS^ (64), K437E (c.1303A>G) ^Mosaic Schnitzler’s syndrome-variant CAPS^ (64, 110), F525L (c.1569C>A; c.1569C>G) ^Mosaic MWS^ (83)^; Mosaic Schnitzler’s syndrome-variant CAPS^ (110), F568L (c.1698C>A) ^Undefined Mosaic CAPS^ (111)^; Mosaic CINCA/NOMID^ (64, 103), E569K (c.1699G>A) ^Undefined Mosaic CAPS^(111), Y572N (c.1708T>A) ^Undefined CAPS with mosaicism^ (64)
Unspecified CAPS/*NLRP3-*AID	H51R (c.146A>G) (64), A77V (c.224C>T) (64) ^+^, R170Q (c.503G>A) (68), V200M (c.562G>A) (5, 68, 70, 112), H215R (c.638A>G) (64), L256M (c.760C>A) ^Undefined atypical CAPS^ (64)^+^, V264G (c.785T>G) (68), G303S (c.901G>A) (64), D305N (c.907G>A; c.913G>A) (5, 69, 71, 76, 89), Q308L (c.917C>T) (66), F311S (c.926T>C; c.932T>C) (90), E313K (c.931G>A) (5), I315V (c.937A>G) (5, 68), S333R (c.993C>A) (5), S334N (c.995G>A) (64) ^+^, I336V (c.1000A>G) (68), T350M (c.1043C>T) (5, 66, 68), P352L (c.1049C>T) (64, 113), V353L (c.1051G>C; c.1051G>T) (64), A354V (c.1055C>T) (66, 68), L355P (c.1058T>C) (68),H360R (c.1073A>G) (66), L371M (c.1105C>A) (5)^; Undefined atypical CAPS^ (64), M408I (c.1218G>C) (5), T438N (c.1307C>A) (66, 71), A441T (c.1315G>A) (66), A441V (c.1316C>T) (5, 66, 68), F446V (c.1330T>G) (64), N479K (c.1431C>A) (5), E527K (c.1573G>A) (68), T544I (c.1630G>A) (64, 114) ^+^, T544M (c.1625C>T) (64, 115, 116), R550C (c.1642C>T) (64), T559A (c.1669A>G) (64), K567E (c.1693A>G) (64), G571R (c.1705G>C) (66, 68, 87), Y572C (c.1709A>G) (66), F575S (c.1718T>C) (66, 87), D648Y (c.1936G>T) (64), L679P (c.2030T>C) (64), E692K (c.2068G>A) (68), M703T (c.2102T>C) (68), Q705K (c.2107C>A) (5, 70, 117, 118), S712C (c.2129C>G) (5), A714S (c.2134G>T) (64), G781V (c.2336G>T) (64, 111), D789N (c.2359G>A) (64), Q798P (c.2387A>C) (64, 104) ^+^, Y861C (c.2576A>G) (68), Y861H (c.2575T>C) (64), G868R (c.2596G>A) (64), S898P (c.2686T>C) (64), R920Q (c.2753G>A) (60, 64, 119), T954M (c.2855C>T) (64), M988I (c.2958G>A) (64) ^+^
Unspecified non-CAPS *NLRP3-*AID	D90Y (c.262G>T) (64), R100G (c.292C>G) (64), R100H (c.293G>A) (64, 90), R178W (c.526C>T) (64, 112), T195K (c.578C>A) (64), E206G (c.611A>G) (64, 120), I290M (c.864C>G) ^Atypical Inflammatory Disease^ (64, 121), R779C (c.2329C>T; c.2335C>T) (64)
Juvenile Idiopathic Arthritis (JIA)	E380K (c.1132G>A) (64, 107), R605G (c.1807A>G) (33)
Rheumatoid Arthritis (RA)	A227V (c.674C>T) (64), M301V (c.895A>G) (64), Q705K (c.2107C>A) (122)
Other and Unknown	D21H (c.55G>C) ^keratoendotheliitis fugax hereditaria^ (64, 123, 124), M70T (c.203T>C) ^UNKNOWN^ (64), T195M (c.578C>T) ^Behcet’s^ (64, 125), V200M (c.562G>A) ^Behcet’s^ (126), P202T (c.598C>A) ^PFAPA^ (64, 127), D282N (c.838G>A) ^UNKNOWN^ (64), I315V (c.937A>G) ^Magic Syndrome^ (64), R327Q (c.974G>A) ^UNKNOWN^ (64), G456E (c.1361G>A) ^UNKNOWN^ (64, 104), I521T (c.1556T>C) ^PFAPA,PFAPA-like^ (64), E640K (c.1912G>A) ^UNKNOWN^ (64, 104), Q705K (c.2107C>A) ^Celiac^ (64)^; PFAPA^ (5)^; UNKNOWN^ (64), G811S (c.2419G>A) ^Atypical Autoinflammatory Syndrome/FMF^ (4), A873T (c.2611G>A) ^UNKNOWN^ (64, 104), N913S (c.2732A>G) ^UNKNOWN^ (64)

^+^Sources that did not clearly identify patients into specific syndromes were reviewed by two clinical authors and grouped into appropriate categories based on presenting symptoms. Patients with a NLRP3 mutation that did not meet criteria for FCAS, MWS, NOMID were placed into Unspecified CAPS/NLRP3-AID.

NLRP3 is composed of an N’-terminal pyrin (PYR) domain, NAIP CIITA HET-E TEP1 (NACHT) domain, and C-terminal leucine-rich repeat (LRR) domains. The PYR domain is responsible for homotypic PYR-PYR interactions with the inflammasome adaptor protein apoptosis speck-like protein containing a CARD (ASC). The NACHT domain senses stimuli through the nucleotide-binding domain (NBD) and is regulated by its regulatory helical 1 (HD1), winged-helix domain (WHD), and helical 2 (HD2) subdomains in addition to the LRR domain, limiting NACHT domain access in the protein’s inactive conformation (129–135). Exon 3 of NLRP3 encodes for its NACHT domain and is known to harbor many mutations associated with both CAPS and non-CAPS *NLRP3-*AIDs.

**Figure 1:**
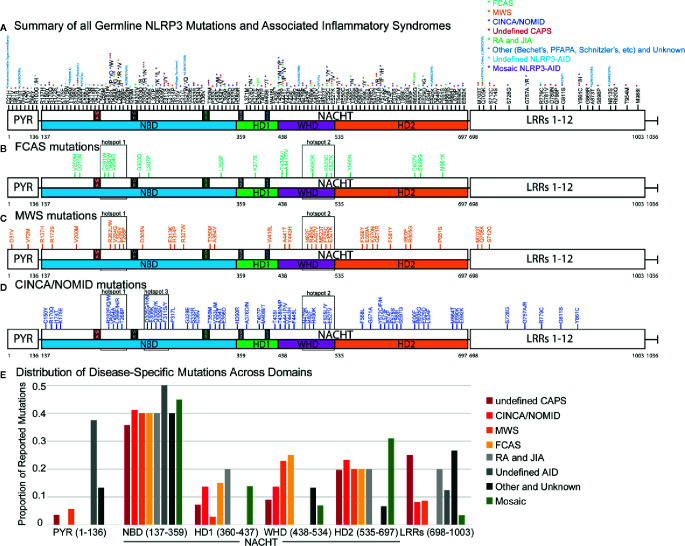
NLRP3 Germline Mutant Protein Maps. **(A)** Schematic representation of NLRP3 mutation proteomic locations known to cause or be associated with cyropyrin-associated periodic syndromes (CAPS) inflammasomopathies [familial cold autoinflammatory syndrome (FCAS), Muckle-Wells Syndrome (MWS), and chronic infantile neurologic cutaneous and articular syndrome, also known as neonatal-onset multisystem inflammatory disease (CINCA/NOMID)], and other *NLRP3-*AID. Annotated at each mutated position is the specific amino acid substitution with notation for the diseases associated with the respective substitution corresponding with the color-coded legend in the top-right corner of the panel. Representative proteomic locations of NLRP3 mutations reported in **(B)** FCAS, **(C)** MWS, and **(D)** CINCA/NOMID. **(E)** The proportion of reported mutations for *NLRP3-*AID and other NLRP3-mutant conditions color-coded in the legend below the graph. NAIP CIITA HET-E TEP1 (NACHT) domain inset annotations: WA, Walker A motif; WB, Walker B motif; S1, Sensor 1 motif; S2, Sensor 2 motif; GxP, GxP motif.

To understand the distribution of disease-specific mutations in *NLRP3*, we plotted all 177 mutations (**Figures 1A–D** and **Supplementary Figure 1**) and analyzed the proportion of mutations between all *NLRP3-*AIDs (**Figure 1E**). We confirmed the high frequency of mutation, 83% (147/177 mutations), in NACHT (**Figure 1A**) (136, 137). In all three CAPS, we found two shared mutational hotspots: the first between the Walker A and Walker B motifs from Cys261 and Thr268, and the second within the WHD between Asn479 to Glu527 (hotspots 1 and 2, **Figures 1B–D**). We identified a third mutational hotspot between Asp305 and Phe311 in CINCA/NOMID (hotspot 3, **Figure 1D**). Among the three CAPS, FCAS mutations exclusively occur within the NACHT domain, while MWS and CINCA/NOMID both have mutations occurring within the LRR domain, and MWS also has mutations in PYR (**Figure 1E**). All CAPS *NLRP3-*AIDs had roughly the same proportion of mutations in the NBD and HD2 subdomains. However, they differed in the WHD subdomain: FCAS and MWS, which are the hereditary and milder CAPS, have a higher proportion of mutations within the WHD subdomain than CINCA/NOMID.

Consistent with other studies (136–138), somatic mosaic mutations occur mostly in the NBD and HD2 subdomains (**Figure 1E**). We confirmed two previously-reported mutational hotspots in the NBD subdomain between Gly303 to Gly309 and within the HD2 domain (136) and identified a third mutational hotspot that straddles the HD1 and WHD subdomains (boxed areas, **Supplementary Figure 1A**). Due to fewer non-CAPS *NLRP3-*AID reports, we were unable to determine whether these *NLRP3-*AIDs had unique mutational hotspots or domain mutational enrichments of significance (**Supplementary Figures 1B–E**).

These data reveal similarities in domain mutation enrichments in all three CAPS, which cannot explain the phenotypic spectrum of CAPS. Therefore, we have decided to investigate other factors that can affect functional changes derived from *NLRP3* mutations.

### Bioinformatics Tools Reveal Inverse Relationship Between Clinical Severity and Structurally Disruptive Potential

The mutations we compiled from the literature lead to a broad range of clinical phenotypes and varying degrees of dysregulation of inflammasome activity, including increased IL-1β cytokine production and decreased monocyte sensitivity to inflammasome-activating triggers such as lipopolysaccharides as observed in CAPS patients (64). Because many of these mutations were reported in the monogenic conditions and considered causative, we speculated that the mutation-related structural changes could alter protein functions and inflammasome activity. While the patterns of mutational lesions have been described (137), no proteomic analyses to date have demonstrated the mechanisms of NACHT domain-driven inflammation or compare each of the CAPS mutations structurally. Multiple distinct factors alter protein function, and specifically for monogenic diseases such as NLRP3-AIDs, these factors include the type of amino acid substitution encoded by genetic changes and where these mutations are located on the protein.

To decipher the differences between CAPS mutations, we used three structural bioinformatics tools: PolyPhen-2 (35), PROVEAN (37, 38), and SIFT (36) to generate functional predictions, i.e., potential to disrupt the structure and function of NLRP3, of all reported mutations. Each tool uses metrics such as sequence homology, sequence length, type of substitution, phylogenetic comparison, structure data, and machine learning in differing combinations to generate tool-specific scores and functional predictions. These tools have occasionally been used in case studies that compare a few mutations at once, but not in conjunction with each other, nor in a large comparative study (83, 139, 140). We combined the scores from these tools to have a reliable scoring metric for mutational analysis, since each utilizes algorithm-specific scoring thresholding parameters. The original outputs of the tools are listed in **Supplementary Table 1**. We ranked all 177 mutations by their combinatorial “p-weighted” scores (details in Materials and Methods). The lower (or more negative if in the log) values for p-weighted scores correspond to more structurally disruptive potential. All combinatorial scores p < 0.05 are shown in **Table 2**. All other combinatorial scores are listed in **Supplementary Table 2**.

**Table 2:** Ranked Combinatorial Scores for NLRP3 Mutants with *p_w_ <*0.05.

Site	Mutation	*p_weighted_*
262	R262W	0.002797268
527	E527V	0.003230007
261	C261W	0.003752929
416	W416L	0.004708504
305	D305A	0.006500263
262	R262L	0.007021087
262	R262P	0.007961164
757	G757R	0.009421334
525	F525C	0.009929316
527	E527K	0.010085693
679	L679P	0.010277819
307	L307P	0.010382365
305	D305G	0.012488896
438	T438I	0.012587706
443	Y443H	0.012587706
438	T438P	0.013202097
954	T954M	0.01688818
634	L634F	0.019636212
305	D305H	0.021691606
21	D21H	0.023883493
305	D305N	0.025728784
303	G303D	0.033630895
306	E306K	0.035895245
334	S334N	0.036482638
408	M408T	0.03648867
661	M661K	0.039405753
262	R262Q	0.041757421
31	D31V	0.042837947
581	F581Y	0.043414953

**Figure 2:**
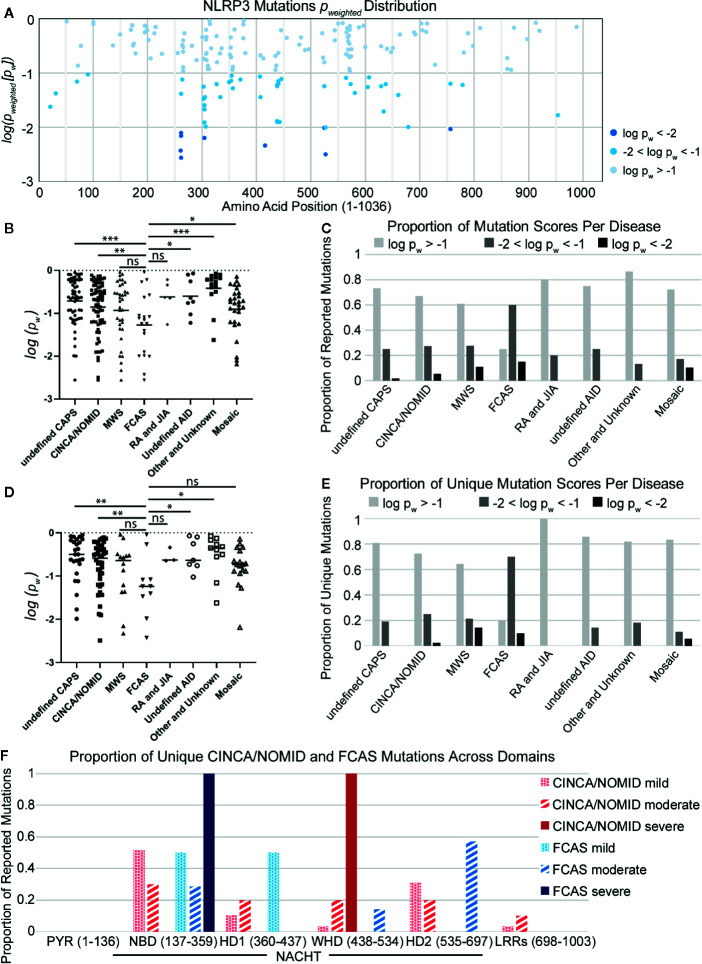
**(A)** Distribution of NLRP3 mutation pw scores across amino acid positions. **(B)** Disease-specific mutations scores. Statistical significance was determined against FCAS by t-test. **(C)** The proportion of reported mutations for *NLRP3*-AID and other *NLRP3*-mutant conditions per *p_w_* score. **(D)** Non-overlapping disease-specific mutation scores. **(E)** The proportion of unique mutations for *NLRP3*-AID and *NLRP3*-mutant conditions per pw score. **(F)** The proportion of unique CINCA/NOMID and FCAS mutations across the *NLRP3* domains per clinical severity (mild – dotted; moderate – striped; severe – solid). Statistical significance was determined against FCAS by t-test. ns, not significant; *p < 0.5, **p < 0.01, ***p < 0.001.

As observed in other studies (136, 141), we found that mutations with the lowest p-weighted scores (log(*p_w_*) < −2, corresponding to *p_w_* < 0.01), which are expected to be the most severely-disruptive to NLRP3 structure and function, occur in the NACHT domain between Glu250 and Arg550 with a mutational hotspot including the Arg262 site (**Figure 2A**). Arg262 is the most cited mutation site (17, 59–61, 64, 66, 69, 93, 96, 136), is shared between all CAPS, and has a wide variance of reported substitutions. The Leu266 and Asp305 sites in this mutational range also have numerous citations (5, 16, 17, 34, 62, 64, 66, 68–71, 76, 90, 93, 97, 108, 142). In contrast, moderately-disruptive mutations (−2 < log(*p_w_*) < −1.3, corresponding to 0.01 < *p_w_* < 0.05) occur not only in the NACHT domain but also in the PYR domain (between Met1 and Arg100) and the LRR domain (between Arg550 and Leu800). Additionally, we observed that most of the mutations we assessed were predicted to have modest changes to the protein structure, which may be in part due to the multimeric nature of the functional NLRP3 inflammasome complex, as the effect of modest changes to the monomer NLRP3 would be multiplied when the multimeric complex is formed. These results demonstrate that severely-disruptive mutations occur within the NACHT domain, whereas moderately-disruptive ones occur in all NLRP3 domains.

To determine whether there is any relationship between the mutations’ disruptive potential and clinical severity, we compared the score distributions of each *NLRP3-*AID. We found that the FCAS had a more negative (i.e., lower) p-weighted score distribution, and therefore was predicted to have more severely-disruptive mutations than most other *NLRP3-*AIDs, including undefined CAPS, CINCA/NOMID, undefined *NLRP3-*AID, other and unknown *NLRP3-*AID, and mosaic *NLRP3-*AID (**Figure 2B**). Further, when the proportion of disruptive mutations was examined in all *NLRP3*-AID, FCAS had the highest proportion of severely-disruptive mutations (log(*p_w_*) < –2) (black bars, **Figure 2C**).

It is tempting to assume that the mutations predicted to be severely-disruptive would correlate with disease severity. However, our data suggest that there may be an inverse relationship between disruptive potential and disease severity. We speculated that large structural changes, such as a large deletion, could lead to non-functional or degraded proteins, whereas the effect of modest changes to the functional NLRP3 proteins would be multiplied when the proteins form the multimeric complex. Alternatively, our observations could be due to overlap between FCAS and a more severe clinical phenotype such as CINCA/NOMID or the mosaic *NLRP3-*AIDs, since many mutations are reported in more than one *NLRP3-*AID.

To address the latter speculation, we reassessed the data using only mutations unique to each condition. We found that the enrichment of structurally-disruptive mutations in FCAS versus CINCA/NOMID remained (**Figure 2D**). Further, FCAS retained the highest proportion of moderately-disruptive mutations among CAPS *NLRP3-*AIDs and still had more severely-disruptive mutations compared to CINCA/NOMID (**Figure 2E**), suggesting an inverse relationship between disruptive potential and disease severity. Further analysis revealed that severely-disruptive and moderately-disruptive mutations of FCAS were enriched in the NBD and HD2 subdomains of NACHT (solid and striped blue bars, respectively, **Figure 2F**). In contrast, for CINCA/NOMID, the severely-disruptive mutations were enriched in the WHD while the moderately-disruptive mutations were distributed relatively evenly across all NACHT domains (solid and striped red bars, respectively, **Figure 2F**).

Together, our analysis of NLRP3-AID mutations demonstrates a surprising inverse relationship between clinical severity and structurally disruptive mutations. We speculated that, for mildly- and moderately-disruptive mutations to correlate with more severe clinical outcomes, there might be biological consequences that our initial analysis of structural changes to NLRP3 was unable to identify. Thus, we decided to investigate severely-disruptive mutations, which have a clear structural impact, to explore their potential mechanisms to alter the normal function of NLRP3.

### Severely-Disruptive NLRP3 Mutations Localize Around ATP Binding Pocket and Alter ATP Binding Affinity

Of the myriad mechanisms regulating the formation of NLRP3 inflammasome (20, 25, 132, 143–149), two critical features are priming and oligomerization. The stimulation-induced ATP priming step activates NLRP3 and helps facilitate conformational changes that mediate adaptor binding (25, 55, 148), whereas oligomerization involves both ASC docking and NLRP3-NLRP3 multimerization (6, 26, 27). Therefore, we explored whether severely-disruptive mutations of FCAS and other CAPS mutations affect these critical molecular mechanisms of NLRP3 activation. To understand their functional consequences, we studied the locations of these mutations in NLRP3 protein.

The cryo-EM structure of NLRP3 has been recently deciphered; however, its relatively low resolution and co-expression with the adaptor protein NEK7 may skew the interpretation of how our mutations affect protein function (132). In order to confidently perform further structural analyses, we generated several structural homology models of human NLRP3 using an established protocol (40) and submitted the protein FASTA to protein modeling servers (41). The top-scoring models were subjected to explicit solvent-based MD simulations (42–45) for refinement (46, 47) and stringent assessment with structure validation tools (48–50), resulting in a refined homology model of quality within one standard deviation of the average values for high-resolution protein crystal structures (further details in the Materials and Methods).

**Figure 3:**
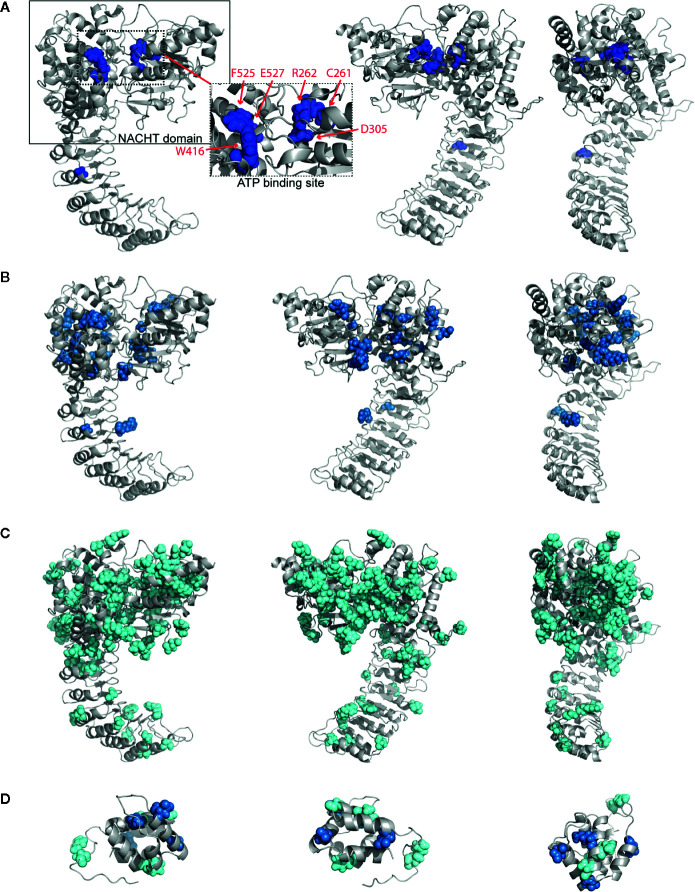
Structurally Disruptive Mutation Sites. Representative locations of **(A)** severely-disruptive mutations (*p_w_* < 0.01; boxes highlight NAIP CIITA HET-E TEP1 (NACHT) domain [solid line] and ATP binding site [dashed line]; inset shows enlarged ATP binding site with labeled mutation sites), **(B)** moderately-disruptive mutations (0.01 < *p_w_* < 0.05), and **(C)** mildly-disruptive mutations (*p_w_* > 0.05) mapped on our NLRP3 homology model, which excludes mutations in the PYR domain and beyond Leu943. **(D)** All germline mutations in PYR mapped on the NLRP3 PYR crystal structure. Severely-disruptive mutations in deep blue, moderately-disruptive mutations in pale blue, mildly-disruptive mutations in cyan spheres.

To examine where the mutations occur and interrogate their biochemical consequences, we plotted all germline mutations onto our refined homology model of NLRP3. We observed a high density of the mutations in and around the NACHT domain (**Supplementary Figure 2**). Nearly 45% of severely-disruptive mutations occur buried within the NBD domain, located explicitly around the ATP binding pocket, while most moderately-disruptive mutations occur along the periphery of the NBD domain instead (**Figures 3A, B**; boxes highlight the NACHT domain and ATP binding pocket). We found that, while these severely-damaging mutations are distributed within separate subdomains, they are indeed close to one another within the ATP binding pocket. These mutations include Cys261, Arg262, and Asp305 within the NBD subdomain, Trp416 within the WHD subdomain, and Phe525 and Glu527 within the WHD subdomain (**Figure 3A**, dashed box).

Since the severely-disruptive mutations are located in the ATP binding pocket (**Figure 3** and **Table 2**), we quantified the mutations on relative ATP binding affinity using small-molecule docking calculations of both ADP and ATP. We analyzed the wild-type (WT) and the top 10 ranked mutations with the lowest p-weighted score, predicted to be structurally severely-disruptive and moderately-disruptive to the protein, and calculated their respective binding energies and the ΔΔG between ATP and ADP. The results are summarized in **Table 3**. While none of the mutations substantively altered the binding affinity for ADP, the majority of the mutations predicted to be disruptive to the protein exhibited an enhanced ATP binding affinity, reflected in lower ΔΔG values.

**Table 3:** ADP, ATP, and ddG Energies for NLRP3 Mutants.

NLRP3	ADP interaction energy (kcal/mol)	ATP interaction energy (kcal/mol)	ΔΔG (ATP-ADP) (kcal/mol)
WT	−9.73	−11.09	−1.36
R262W	−9.54	−11.69	−2.15
C261W*	−9.61	−11.37	−1.76
E527V^#^	−9.58	−11.99	−2.41
W416L^+^	−9.83	−11.20	−1.37
D305A	−9.68	−11.13	−1.45
R262L^+^	−9.79	−12.09	−2.30
R262P	−9.68	−12.38	−2.70
G757R	−9.67	−10.98	−1.31
F525C	−9.73	−11.07	−1.34
E527K	−9.28	−11.91	−2.63

Phenotype specificity: (*) FCAS, (^+^) MWS, (^#^) CINCA/NOMID.

We note that this computational modeling has the limitation of being derived from a homology model, and the extent to which the ATP binding enhancement occurs may be different when tested in the laboratory. Nonetheless, our data suggest that one mechanism of the severely-disruptive mutations is *via* enhanced ATP binding affinity, resulting in a greater propensity of these mutants for NLRP3 activation. The data also suggest that the structural bioinformatics tools highlight differences in protein-intrinsic function, such as regulation of NLRP3 activation by ATP priming.

### CAPS Mutations Differentially Enhance ATP Binding by Altering Hydrogen-Bond and Charge Interactions

**Figure 4:**
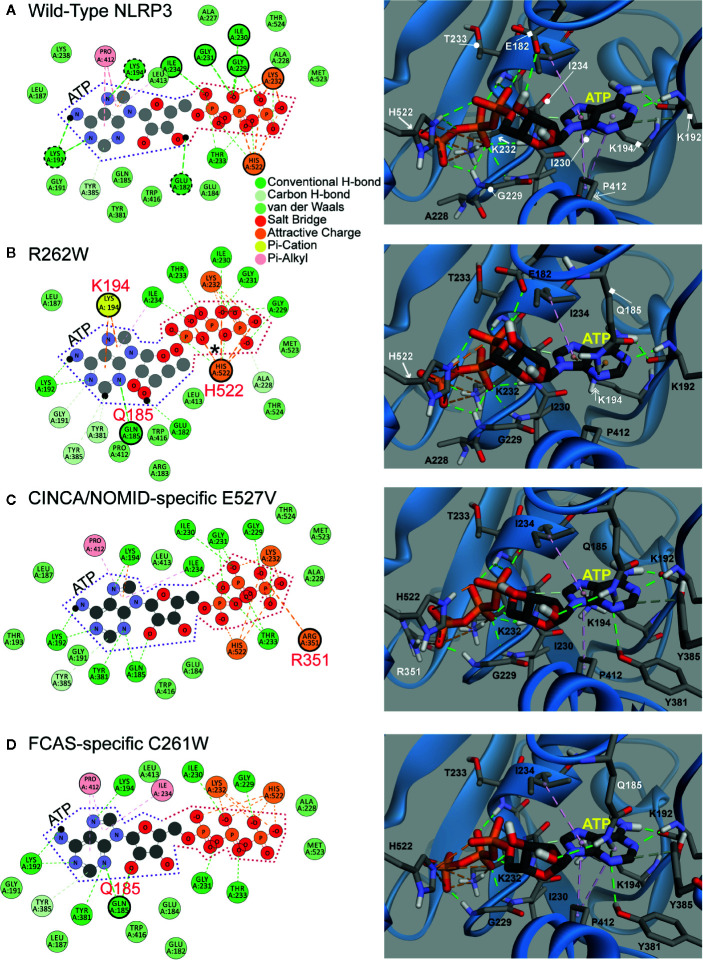
2D and 3D Interaction Plots of Mutations Affecting ATP Binding. **(A, B)** 2D and 3D Interaction Plots of wild-type) (WT) NLRP3 and the R262W mutant. **(C, D)** 2D and 3D interaction plots of chronic infantile neurologic cutaneous and articular syndrome, also known as neonatal-onset multisystem inflammatory disease (CINCA/NOMID)-specific E527V mutant and familial cold autoinflammatory syndrome (FCAS)-specific C261W mutant. ATP is demarcated by purple and orange dotted lines in the 2D panel (left panels). In the 3D panels (right panels), ATP is shown in a stick model with the yellow label, while NLRP3 is shown as a ribbon model.

Next, to understand how ATP priming is facilitated, we analyzed the ATP binding pocket of WT NLRP3 and the top three most structurally disruptive mutations: R262W, C261W, and E527V, which are also the most prevalent in CAPS (**Figure 4**). ATP is demarcated by dotted lines in the 2D panels (**Figure 4**, left panels): purple dotted lines surround adenosine, and orange dotted lines surround the triphosphate. In the 3D panels of **Figure 4** (right panels), ATP is the stick model with the yellow label, while NLRP3 is in the ribbon model.

Within the binding pocket of the WT NBD (**Figure 4A**), ATP is stabilized by the interactions with Leu232 and His522 (orange circles and arrows in the 2D and 3D panels, respectively). H-bonds (green dashed lines) from Ile234, Ile230, Gly231 (out of plane), Gly229, and Thr233 also stabilize ATP on the triphosphate (green circles and circle-ended lines in the 2D and 3D panels, respectively). Furthermore, adenosine is stabilized by H-bonds with the surrounding residues Lys192, Lys194, and Glu182 (dashed green circles and square-ended lines in the 2D and 3D panels, respectively), and hydrophobic interactions (pink dashed lines) with Pro412 (pink circle and double arrow in the 2D and 3D panels, respectively).

We compared the models between WT NLRP3 and that of R262W (**Figure 4B**). The R262W mutation shifts the triphosphate- and adenosine-stabilizing interactions to enhance ATP binding. Due to the loss of positive charge and bulky substitution of tryptophan in the R262W mutation, the site no longer participates in the direct interactions with ATP within the binding pocket (R262W is beyond the plane of view). For the triphosphate, an additional attractive interaction (marked by an asterisk in the 2D panel) occurs on the first phosphate group from His522 (orange circle and arrow in the 2D and 3D panels, respectively). For the adenosine, Gln185 (green circle and square-ended line in the 2D and 3D panels, respectively) now H-bonds (green dashed lines) with adenine ring, and the pi-stacking interaction (orange dashed lines) with Lys194 (yellow circle and double arrow in the 2D and 3D panels, respectively) is stronger and more prominent than in the WT, resulting in more favorable binding with ATP.

Together, the modeling of R262W revealed that the substitution of tryptophan in the 262 site likely replaced a positive charge with an uncharged and bulky amino acid, changing the interactions between ATP and the ATP pocket to more favorable and attractive interactions between ATP and other amino acids, including histidine, glutamine, and lysine. These favorable bindings with ATP, together with higher baseline levels of autocrine ATP observed from CAPS monocytes, may explain why there is more activation of the NLRP3 inflammasome and IL-1β secretion in CAPS patients (20).

We then explored the impact of the second and third most structurally disruptive mutations identified in our bioinformatics analyses: E527V and C261W (**Figures 4C, D**). Incidentally, these mutations are specific to CINCA/NOMID and FCAS, respectively. In the WT structure, Glu527 is involved in a salt bridge with Arg351, while Cys261 does not directly interact with ATP (all three sites are outside of the plane of view in **Figure 4A**). The E527V mutation abrogates the aforementioned ionic interaction: the Glu527-Arg351 salt bridge, and alters the positioning of Arg351 to farther into the ATP binding site where it interacts with terminal phosphate of ATP directly, thus enhancing binding (**Figure 4C**). Similarly, the C261W mutation alters the binding pocket as it adds two hydrogen bond interactions with Gln185 and decreases the intermolecular distance between the terminal phosphate and the basic residues in this region of the binding site (**Figure 4D**).

Similar to the computational modeling of R262W, the study of the second and third most structurally disruptive mutations, E527V and C261W, revealed that substitutions at these sites likely altered the ATP binding pocket interactions such that ATP more favorably interacts with Arginine (for E527V) and glutamine (for C261W). Further laboratory study is needed to verify how these three mutations affect ATP binding.

Next, we extended our examination of ATP binding enhancement through adding or shifting stabilizing interactions to a group of 10 FCAS-specific and 40 CINCA/NOMID-specific mutations in an attempt to determine whether either group exhibited greater dependency on enhanced ATP binding or other potential biochemical mechanisms to enhance NLRP3 activation. Our observations are summarized in **Supplementary Table 3**. Among 10 FCAS-specific mutations, three (C261W, L307P, and G303D) showed differences in ATP binding affinity compared to WT (ΔΔG column, **Supplementary Table 3**). Two FCAS mutations in proximity to the ATP binding site, L307P and G303D (both are out of the plane in **Supplementary Figures 3A, B**), had a much stronger effect on ATP binding than C261W, and resulted in repositioning to move the residue further into the ATP site and directly interacting with the terminal phosphate of ATP, suggesting that all three among 10 FCAS-specific mutations enhance ATP binding.

On the other hand, four (E527V, D305G, E306K, and R262Q) out of 40 CINCA/NOMID-specific mutations had differences in ATP binding affinity compared to WT (ΔΔG column, **Supplementary Table 3**). E306K exhibits enhanced ATP binding affinity (**Supplementary Figure 3C**). However, unlike the other ATP affinity-enhancing mutations, E306K is shifted into the pocket to directly interact with ATP’s terminal phosphate (marked with a black asterisk), doubly enhancing ATP’s binding affinity (**Supplementary Table 3**). E306K also strengthens the pi-stacking interaction with Lys194 (yellow circle), repositions Arg262 to stabilize the terminal phosphate (orange circle marked with red arrow), and shuffles the H-bonds on both the adenosine ring and the triphosphate compared to WT (green dashed lines and green asterisks). However, CINCA/NOMID-specific D305G and R262Q do not appear to significantly affect ATP vs ADP binding affinity (**Supplementary Table 3**), demonstrating that only two among the 40 CINCA/NOMID-specific mutations enhance ATP binding.

Overall, the computational modeling of NLRP3 predicts enhanced ATP binding as a mechanism of NLRP3 activation in CAPS, indicating that enhanced ATP binding and increased susceptibility to activation may be one of the primary drivers. While some findings suggest that the FCAS mutants are more likely to enhance ATP binding affinity to a higher degree than mutations specific to CINCA/NOMID, further study is necessary to verify this observation experimentally.

### CAPS Mutations Enhance NLRP3-ASC Binding and NLRP3 Multimerization

Based on the bioinformatics tools, the PYR domain mutations are predicted to be either moderately- or mildly-disruptive, but the bioinformatics tools do not integrate protein-protein interaction disruption into their algorithms. The inter-protein interactions that stabilize inflammasome formation and activity may be significantly affected by the mutations, which yet remain unexplored.

We hypothesized that PYR mutations might potentially enhance NLRP3-ASC binding and facilitate inflammasome formation. We identified nine PYR mutations to evaluate: D31V and V72M from MWS (**Figure 1C**); H51R and A77V from undefined CAPS (**Supplementary Figure 1B**); D90Y, R100G and R100H in undefined *NLRP3-*AID (**Supplementary Figure 1C**); and D21H and M70T from other *NLRP3-*AID (**Supplementary Figure 1E**). As of this writing, these mutations have not been characterized for NLRP3 oligomerization. We performed two computational modeling analyses and examined mutations that might possibly affect NLRP3-ASC binding to explore their potential mechanisms.

**Figure 5:**
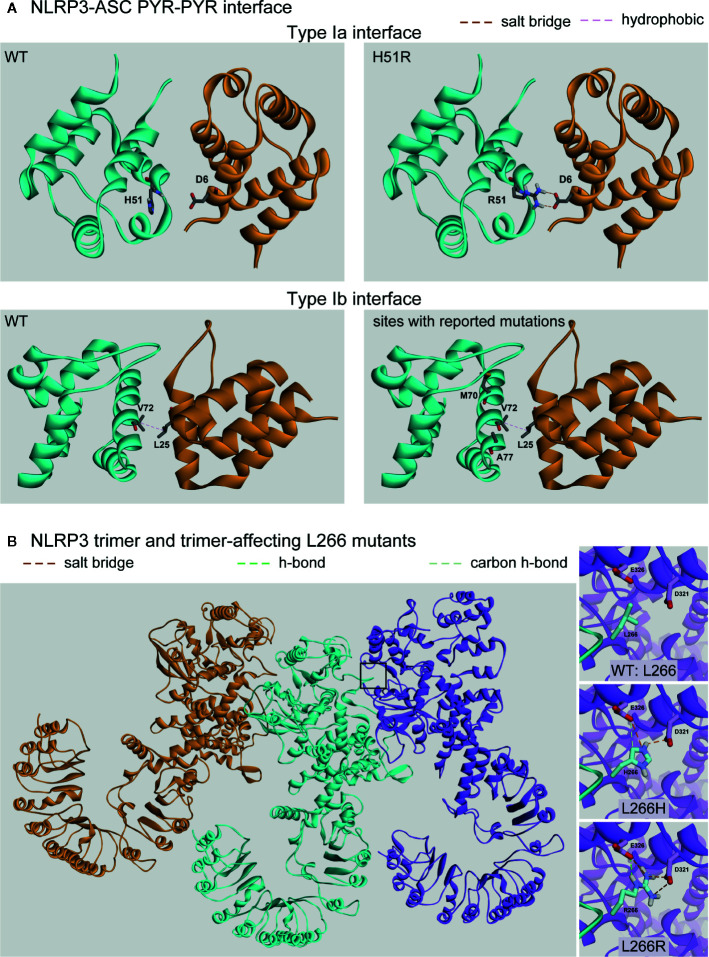
Protein-Protein Affecting Mutations. **(A)** Predicted interaction between the pyrin domains of NLRP3 (cyan) and ASC (orange) at either the Type Ia or Type Ib interfaces. **(B)** The trimer of NLRP3 monomers (orange, cyan, and purple) with the predicted intermolecular interactions between the wild-type (WT) (Leu266) or the two identified mutations (L266H and L266R).

The NLRP3 inflammasome adaptor ASC is bipartite, having an N’-terminal PYR and C’-terminal CARD. ASC binds the PYR domain of NLRP3 through a homotypic domain interaction (55, 148, 150) and the inflammasome effector CASP1 through another homotypic interaction *via* its CARD domain. We performed protein-protein docking studies to examine the interactions between the PYR domains of NLRP3 and ASC. The domains associate across two different interfaces of ASC, one involving helices 1 and 4 (type Ia interface; **Figure 5A**, top) and the other involving helices 2 and 3 (type Ib interface; **Figure 5A**, bottom) (150). Our results with WT NLRP3 are in good agreement with those previously reported (55).

Of the nine mutations reported in the PYR domain, we found four mutations are present at one of these two interfaces with the potential to directly affect the intermolecular interactions: H51R, M70T, V72M, and A77V. However, only the H51R mutation was predicted to alter the interaction pattern, which introduced a salt-bridge interaction between Arg51 on NLRP3 with Asp6 on ASC (**Figure 5A** top-right), resulting in an enhanced binding between PYRs by 2.8 kcal/mol. The rest of the tested mutations had no direct effect on the interactions, and due to the limitations of the NMR structure (55), we did not model the R100G or R100H mutations. While laboratory experiments are needed to verify enhanced binding, among the four mutations occurring at the NLRP3-ASC interaction surface, our study revealed that only H51R was predicted to result in enhanced binding between the PYR domains of both proteins.

We then hypothesized that some mutations in the NACHT domain could affect NLRP3-NLRP3 multimerization, which is also vital for inflammasome formation. Therefore, in addition to the extended examination for changes to ATP binding potential for the group of FCAS-specific and CINCA/NOMID-specific mutants, we evaluated whether these mutations would affect NLRP3-NLRP3 binding (Evaluation Notes column, **Supplementary Table 3**). The NLRP3 inflammasome is believed to form a similar structure to the NLRC4-NAIP2 inflammasome (151), which was resolved as an 11-mer (1 NAIP2:10 NLRC4 monomers) complex experimentally. Thus, mutations that energetically affect NLRP3-NLRP3 oligomerization would be magnified 10-fold and could profoundly affect inflammasome formation and activity.

To examine which mutations are located at the interaction interface between NLRP3 monomers in the assembled inflammasome complex, we generated a trimeric NLRP structure based on that of the NAIP2-NLRC4 inflammasome complex (**Figure 5B**) (151). We found that two mutations unique to CINCA/NOMID at position 266 (L266H and L266R) are located at the interaction interface. In both cases, each mutant induced the formation of two additional salt bridges with Asp321 and Glu326 of the neighboring monomer (**Figure 5B** insets). We found that these interactions enhanced the monomers’ binding affinity by 3.4 and 4.2 kcal/mol for the L266H and L266R mutants, respectively. An inflammasome complex containing ten mutants would therefore exhibit a substantial gain in total interaction energy of 34 and 42 kcal/mol, respectively, that would also translate to the greater overall stability of the oligomeric complex versus one containing only WT monomers.

Altogether, the computational modeling of NLRP3 identified enhanced inflammasome formation and complex stability as a mechanism of NLRP3 activation in CAPS. Further study is necessary to verify this observation experimentally.

## Discussion

Though NLRP3 is the common denominator of all NLRP3-AIDs, the differences in clinical manifestations and disease onset distinguish them from each other. We identified both mutational hotspots and unique regions where disease-specific mutations occur. CAPS generally report mutations in similar regions of the NACHT domain, while non-CAPS have no apparent pattern. Among CAPS, FCAS mutations exclusively occur within the NACHT domain, MWS uniquely has mutations within the PYR domain while FCAS and CINCA/NOMID do not, and the mutational hotspot between Asp305 and Pro317 seems unique to CINCA/NOMID. By modeling and comparing sets of FCAS-specific and CINCA/NOMID-specific mutations, we found that enhanced ATP binding might be a primary driver toward an enhanced priming state. We also found mutation-enhanced protein-protein interactions as another mechanism to enhance inflammasome formation and complex stability.

Using bioinformatics tools and computational modeling, we demonstrated that disruptive mutations are enriched in the NACHT domain, specifically within the ATP binding pocket, whereas moderately-disruptive mutations are localized outside of the ATP binding pocket. We confirmed that mutations farther away from the ATP binding pocket, even within the NBD, had little bearing on nucleotide (ATP/ADP) binding affinity. Notably, our analysis revealed an inverse relationship between clinical severity and predicted mutation severity: the clinically severe phenotype, CINCA/NOMID, was attributed mostly to moderately-disruptive mutations while the clinically mild phenotype, FCAS, was attributed mostly to severely-disruptive mutations. The severely-disruptive mutations of FCAS were enriched in the NBD and HD2 subdomains. Although they were at opposite ends of NACHT, our computational modeling shows that they were indeed close to one another within the ATP binding pocket and played an essential regulatory role in controlling ATP binding and access to the binding pocket. On the other hand, severely-disruptive mutations in CINCA/NOMID were enriched in the WHD subdomain, whose histidine residues interact mostly with the phosphates of nucleotide substrates such as ATP and whose structure is thought to impose specific conformation on the neighboring HD2 domain to lock NLRP3 in an inactive state (152). Although the inverse relationship statistically stands out, we expect that complex biology drives it, which may not be exclusively explained by mutation-induced structural changes. Our data examining R262W is an excellent example of this complexity, where the same mutation is responsible for causing each distinct CAPS phenotype; therefore, there must be more factors affecting the clinical phenotypes.

Notable modern scoring tools such as Functional Analysis through Hidden Markov Models (FATHMM) (153) and Combined Annotation-Dependent Depletion (CADD) (154) have not been used in case reports or databases mined for variants included in this study. However, the reliability and track record of these tools makes them appealing to use in addition to our combined scoring system. Therefore, as a comparison, we first used FATHMM to score all 177 mutations (**Supplementary Table 4**). We compared the 29 variants from **Table 2** and the top 29 variants ranked by their FATHMM score and found 8/29 shared hits: E527V, D305A, F525C, E527K, D305G, L634F, D305H, and M661K. In contrast to the top three variants selected by our method, R262W, E527V, and C261W, the top three variants selected by FATHMM were F525C/Y/L, indicating more structural and functional impacts all occur at the Phe525 site. These three and the following variants R490K, G571R, I521T, E569A, E569K, G571A, and T544M comprise the top ten FATHMM selected structurally-disruptive mutations and are located peripherally to the ATP binding site within the WHD and HD2 domains. While experimentally unconfirmed, it appears that the FATHMM scores highlight the regulatory WHD and HD2 domains preferentially over mutations directly affecting the ATP binding pocket.

Next, we used CADD. Due to the CADD’s specific formatting requirement, limitations to our gathered variant information, and mismatch of the genetic information encoding many of these NLRP3 proteomic variants, we only analyzed the 46 mutations we could obtain from gnomAD (39) (**Supplementary Table 4**). The PHRED CADD scores were normalized to ~9 billion SNVs, and variants scoring 20 or greater indicate that the Raw CADD score was in the top 1%, giving us confidence that these are likely disruptive mutations (154). The top eight mutants with a PHRED CADD score above 20 were T954M, D21H, M70T, R779C, E380K, E607V, and E327W and R605G. The top two mutations, T954M and D21H, were included in **Table 2**. In contrast to FATHMM and our combined scoring system, there does not seem to be a domain or subdomain preference highlighted by CADD. Unfortunately, the T954M variant is outside of our homology model’s limits and may affect either the oligomerization of the protein or the autoinhibition exerted by the LRR domain. The two PYR mutations D21H and M70T were examined in Section 4.5. In addition to scoring the remaining 131 variants that we could not score using CADD, experiments are needed to confirm any of the predictions examined using these algorithms.

NLRP3 function has been tied to cAMP, levels of reactive oxygen species (ROS), and redox states, among others, and we understand that mutations in NLRP3 can affect these functions (20, 34, 146, 155). For example, one study determined that the NBD of NLRP3 mediates cAMP binding and that the binding affinity of WT NLRP3 for cAMP was substantially higher than that of CAPS mutants D305N, A354V, and F525C (146). As an inactivator of the NLRP3 inflammasome, changes in cAMP levels or cAMP binding affinity may underscore the difference in activation threshold between CAPS mutant variants of NLRP3 and WT. Another found that the monocytes of a MWS patient carrying the R262W mutant produced the highest amounts of secreted IL-1β with the fastest secretion kinetics compared to both healthy donor monocytes and even CINCA/NOMID monocytes (20). Correspondingly, higher baseline levels of ROS were present in the MWS patient monocytes as well. One more study compared the redox states between two related patients, a father and a daughter, carrying the T350M mutation presenting with MWS and MWS/CINCA overlap, respectively. The redox states were correlated with disease severity, and MWS/CINCA overlap monocytes had higher IL-1β secretion, lower activation threshold, higher levels of ATP secretion, and more impaired antioxidant response than the MWS monocytes (155). While their results seem contradictory to the correlation we observe, the T350M mutation had mixed results when evaluated bioinformatically (**Supplementary Table 1**), similar to the mixed biological differences found in the previous study. Further cases testing for redox states and responses between nonoverlap CAPS are needed to correlate altered redox states with disease severity definitively. Beyond comparing the two related patients, their study also found that the production of IL-1Rα, which inactivates the IL-1β-driven inflammatory response, is impaired in CAPS (155). These studies show CAPS monocytes exhibit not only higher baseline ROS, ATP release, and IL-1β secretion but also a lower activation threshold resulting in faster IL-1β secretion kinetics in response to lipopolysaccharide stimulation concurrent with a depressed oxidative response compared to WT monocytes (20, 34, 155). Thus, molecular experiments are necessary to definitively determine why we observed more structurally disruptive mutations in the least clinically severe CAPS and, conversely, why we observed less structurally disruptive mutations in the most clinically severe CAPS.

Given that the mutation is a dominant condition, the inflammasome complexes could consist of a heterogeneous population of WT and mutant monomers. Because residue L266 is located on the periphery of the NLRP3 protein, it makes no intramolecular contacts with other residues in either the WT or mutant state. Therefore, the mutations did not affect the protein region containing D321 and E326 (**Figure 5B** insets, a comparison of three structures), and the neighboring monomer’s interaction interface was identical, no matter whether L266 was a mutant or WT. When an inflammasome complex contains a mixed population of monomers, the total gain in binding affinity and complex stability would be the gain of each monomer pair multiplied by the number of mutant monomers in the complex.

While protein-protein interactions between ASC-NLRP3 and NLRP3-multimers are essential for forming large complexes such as the inflammasome, the scores for mutations using our structural bioinformatics tools were either moderately-disruptive or mildly-disruptive and did not concur with the impact of enhanced binding affinity we observed when multiplied by the number of expected monomers in the fully constituted inflammasome complex. This may be explained by differences in the mutant monomers’ expression and the potential heterogeneity of the various inflammasome complexes to the number of mutant NLRP3 monomers contained therein. Our results certainly justify a further review of additional activation mechanisms for complexes such as the inflammasome.

The modeling studies described here are not intended to serve as a comprehensive description of each mutation’s mechanism but rather to examine the effects of selected mutations located in critical regions of NLPR3 and tease apart the potential mechanism(s) playing a role in disease. The findings support the hypothesis that some of the mutations may result in enhanced activity or a greater propensity to activate inflammasome formation. However, this does not preclude additional mechanisms or additional indirect effects of the other mutations responsible for the observed clinical phenotypes. For example, mutations in the hinge region or LRR may result in loss of regulatory inhibition or constitutive activation of NLRP3. Future studies will delve further into the molecular mechanics of additional mutations not described here.

While only two of the PYR mutations we identified were from MWS, the remaining PYR mutants from undefined CAPS and undefined *NLRP3-*AID may either be undiagnosed MWS or share clinical manifestations with MWS. If true, the mutation-enhanced NLRP3-ASC interactions may be specific to MWS or MWS-like cases. So far, our analysis only identified mutation-enhanced NLRP3-NLRP3 interactions in two CINCA/NOMID mutations, but more mutants may enhance these interactions under dynamic conditions. Additional studies are necessary to evaluate this possibility. Further, these mechanisms may be specific to CAPS, given some evidence that JIA and non-CAPS NLRP3-AID do not exhibit similar redox remodeling as CAPS (20). To that end, other non-CAPS AID such as Bechet’s and PFAPA may have unique remodeling, and thus further study is needed to clarify which mechanisms differentiate not only CAPS but each NLRP3-AID.

In summary, in the current paper, we identified structurally stabilizing interactions of NLRP3, which enhance ATP binding and are likely to lower the activation threshold for inflammasome formation and activity observed in CAPS. Another potential mechanism we identified in CAPS pathogenesis is enhancing various protein-protein interactions between NLRP3 oligomers or between NLRP3 and ASC in the assembled inflammasome complex. These enhanced interactions can favor the formation and/or stability of the complex as a whole and may ultimately result in a greater propensity for inflammasome activation. Although our modeling efforts did not identify a functional role for all of the identified mutations, it is possible that under dynamic conditions, these mutations may have an indirect effect of the protein structure that likewise results in gain-of-function or impaired negative regulation of the inflammasome. Further MD studies are necessary to parse out the structural and functional implications of the other mutations not assessed in the current study, particularly those localized to the hinge and LRR domains, and will be a focus of future research efforts.

## Data Availability Statement

The raw data supporting the conclusions of this article will be made available by the authors, without undue reservation.

## Author Contributions

Funding acquisition, MF. Conceptualization, MF, JS, DB. Supervision, MF. Experimentation, JS and DB. Formal analysis, JS, DB, PV, NK, DR, ZZ, JD, and MF. Writing—original draft preparation, JS and DB. Writing—review and editing, JS, DB, PV, NK, DR, ZZ, JD, and MF. All authors contributed to the article and approved the submitted version.

## Funding

This work has been supported, in whole or in part, by Veterans Affairs Merit Review Award 5I01BX001228 (to MF), NIH/NCI R01 CA197919 (to MF), and Cancer League of Colorado (to MF).

## Conflict of Interest

The authors declare that the research was conducted in the absence of any commercial or financial relationships that could be construed as a potential conflict of interest.
