# Supplementary material for: Computational Modeling of NLRP3 Identifies Enhanced ATP Binding and Multimerization in Cryopyrin-Associated Periodic Syndromes
Source: Front Immunol. 2020 Nov 19;11:584364. doi: 10.3389/fimmu.2020.584364 (PMC7711157; doi:10.3389/fimmu.2020.584364)
Supplement: Supplementary file 1 [file DataSheet_1.docx]

Supplementary Material


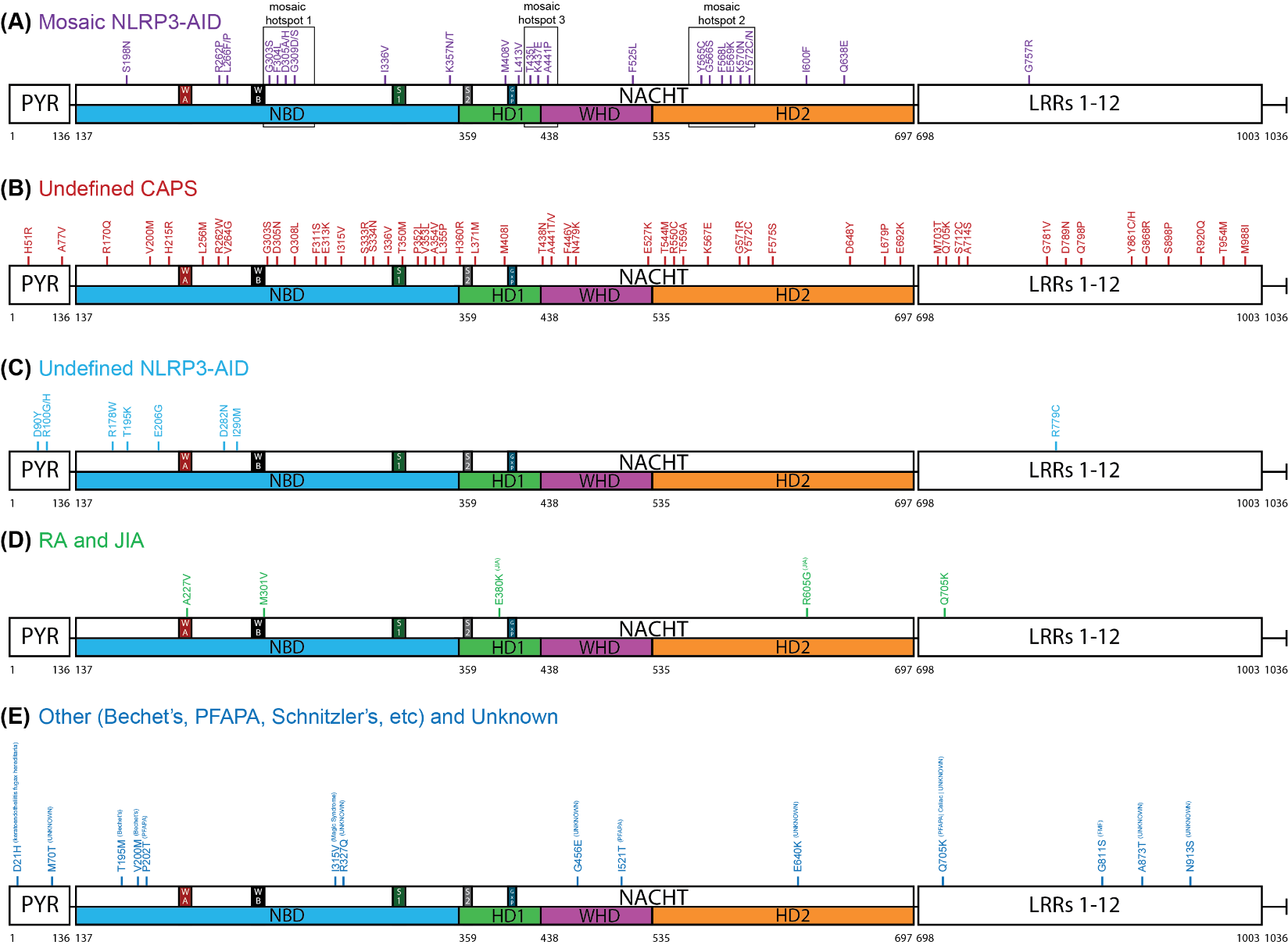


**Supplementary Figure 1. NLRP3 Germline Mutant Protein Maps from Additional Diseases**
Schematic representation of NLRP3 mutations proteomic locations reported in (A) Mosaic *NLRP3-*AID, (B) Undefined CAPS, (C) Undefined *NLRP3-*AID, (D) RA and JIA, and (E) Other and Unknown.


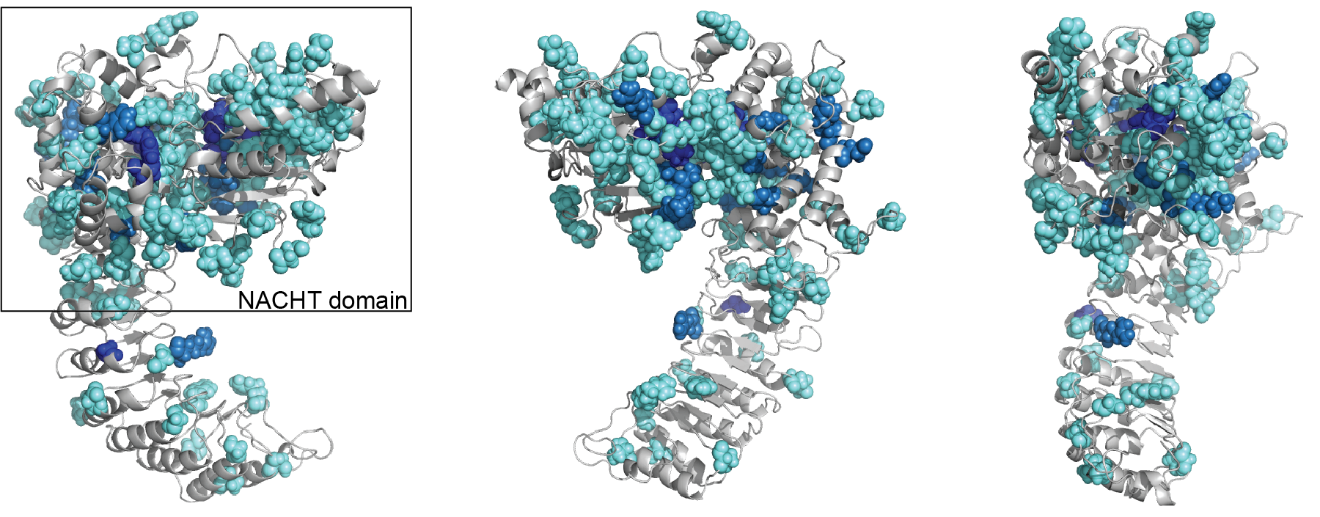


**Supplementary Figure 2. Additional NLRP3 Mutation Plot**

Representative locations of all germline mutations mapped on our homology model which excludes mutations in the PYR domain and mutations beyond Leu943.


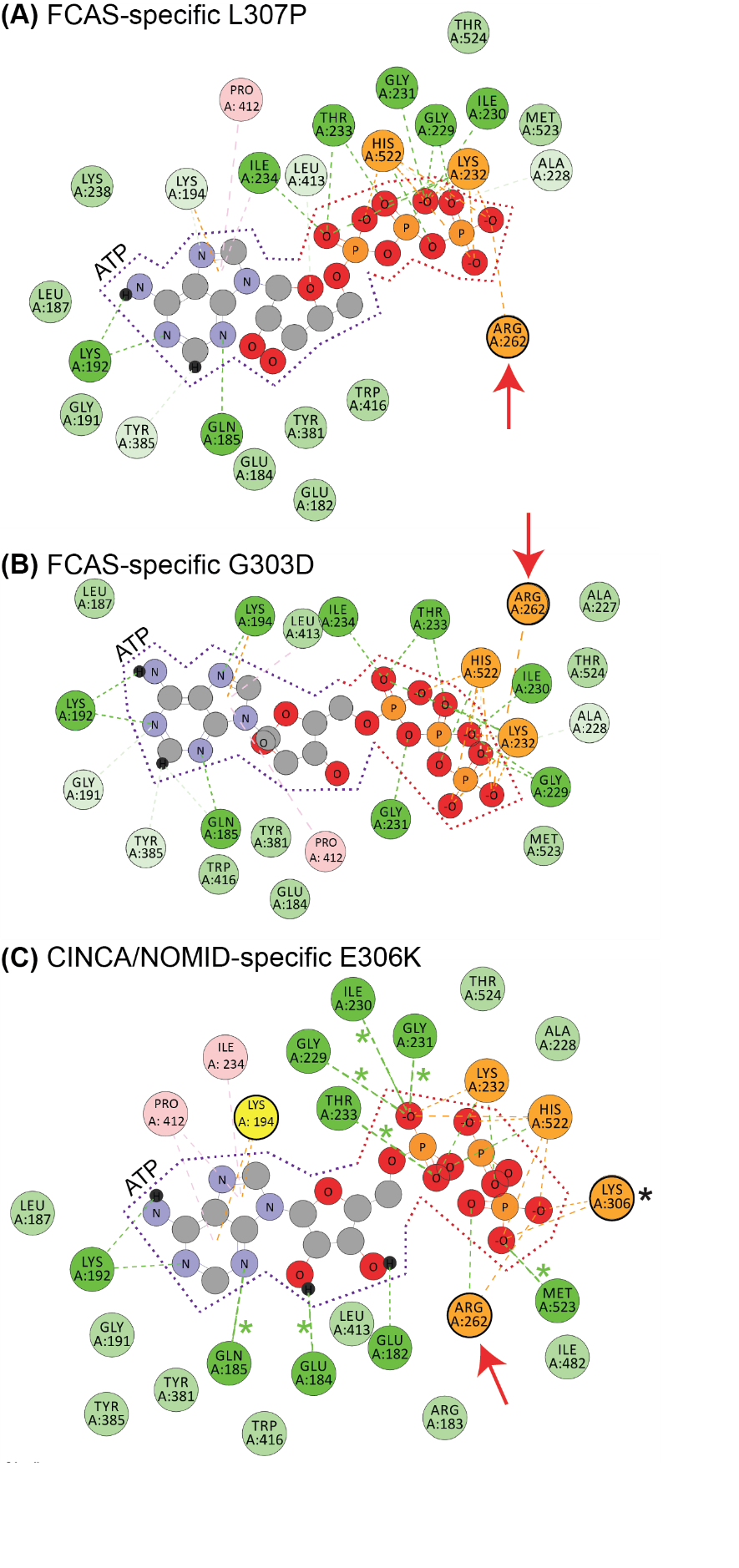


**Supplementary Figure 3. Additional ATP Binding 2D Interaction Plots**2D Interaction plots of ATP binding affinity enhancing mutants (A) L307P, (B) G303D and (C) E306K. Black * indicates where the mutation itself is involved in stabilizing ATP; Green * indicate where shifted or new H-bonds occur compared to WT. Arg262 is marked with red arrows.


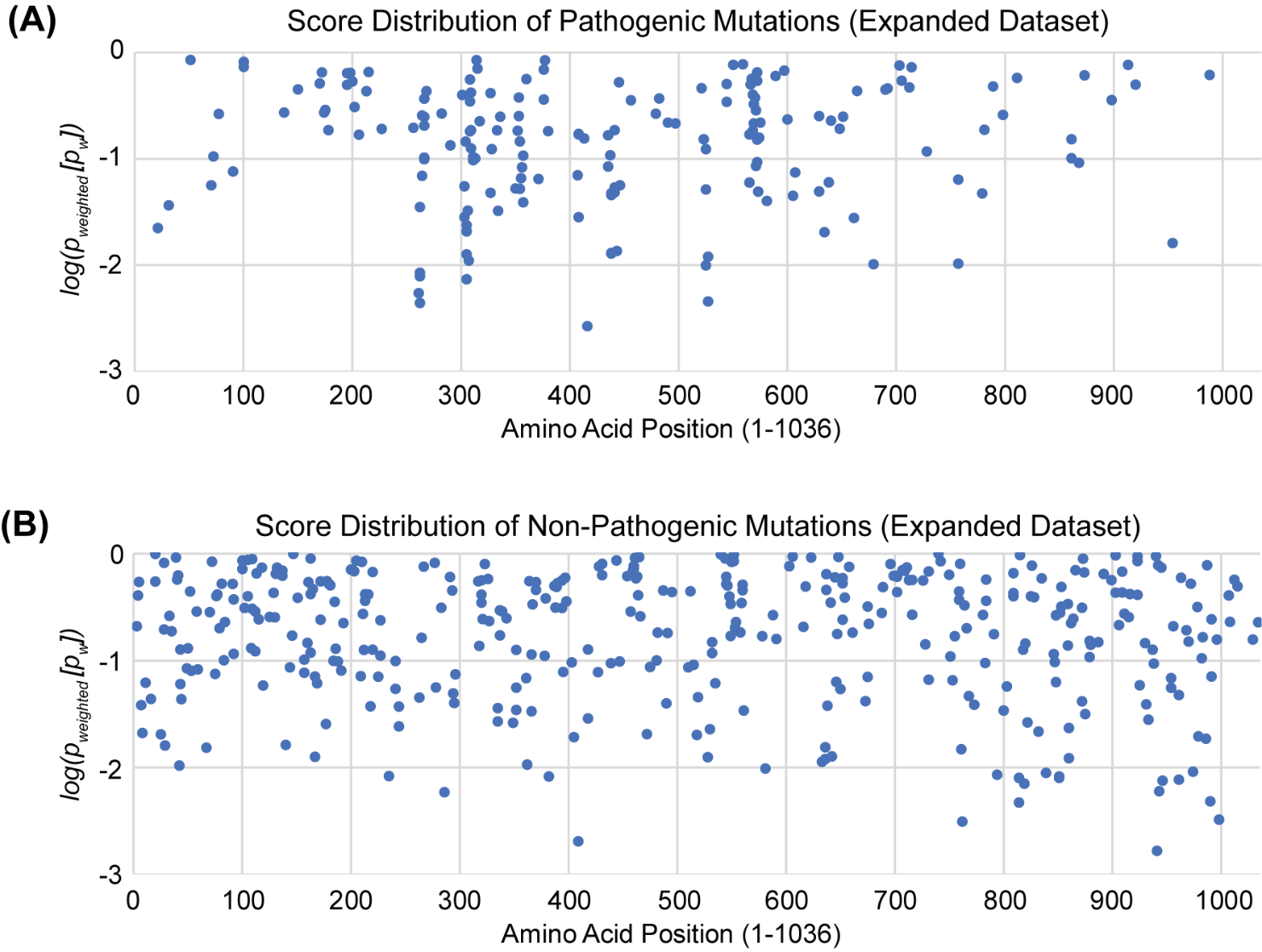


**Supplementary Figure 4. Additional Distribution Plots**(A) Distribution of pathogenic NLRP3 mutation *p_w_* scores across amino acid positions. (B) Distribution of non-pathogenic, rare NLRP3 single-nucleotide variant (minor allele frequency < 0.001) *p_w_* scores across amino acid positions.

| **Supplementary Table 1: Bioinformatics Scores of All Reported NLRP3 Germline Mutations** | | | | | | |
| --- | --- | --- | --- | --- | --- | --- |
|  |  |  |  |  |  |  |
| Mutation | PPH2 Score | **PolyPhen2  Predictions^a^** | PROVEAN Score | **PROVEAN  Prediction^b^** | SIFT Score | **SIFT  Prediction^c^** |
| W416L | 1 | **probablydamaging** | -11.16 | **Deleterious** | 0.033 | **Damaging** |
| C261W | 1 | **probablydamaging** | -7.405 | **Deleterious** | 0.001 | **Damaging** |
| R262W | 1 | **probablydamaging** | -7.022 | **Deleterious** | 0 | **Damaging** |
| E527V | 1 | **probablydamaging** | -7 | **Deleterious** | 0 | **Damaging** |
| L307P | 0.988 | **probablydamaging** | -6.395 | **Deleterious** | 0 | **Damaging** |
| D305A | 1 | **probablydamaging** | -6.255 | **Deleterious** | 0.001 | **Damaging** |
| R262L | 1 | **probablydamaging** | -6.124 | **Deleterious** | 0.001 | **Damaging** |
| G757R | 0.997 | **probablydamaging** | -6.035 | **Deleterious** | 0 | **Damaging** |
| R262P | 1 | **probablydamaging** | -6.024 | **Deleterious** | 0.001 | **Damaging** |
| T438I | 0.999 | **probablydamaging** | -5.767 | **Deleterious** | 0.001 | **Damaging** |
| T438P | 0.999 | **probablydamaging** | -5.7 | **Deleterious** | 0.001 | **Damaging** |
| Y572C | 0.162 | **benign** | -5.69 | **Deleterious** | 0.024 | **Damaging** |
| D305G | 1 | **probablydamaging** | -5.595 | **Deleterious** | 0.004 | **Damaging** |
| F525C | 1 | **probablydamaging** | -5.505 | **Deleterious** | 0.001 | **Damaging** |
| L679P | 1 | **probablydamaging** | -5.426 | **Deleterious** | 0.001 | **Damaging** |
| E206G | 0.001 | **benign** | -5.125 | **Deleterious** | 0.016 | **Damaging** |
| T954M | 0.999 | **probablydamaging** | -5.113 | **Deleterious** | 0.001 | **Damaging** |
| V264G | 0.418 | **benign** | -5.055 | **Deleterious** | 0.002 | **Damaging** |
| E607V | 0.382 | **benign** | -5.032 | **Deleterious** | 0.002 | **Damaging** |
| E629G | 0.768 | **possiblydamaging** | -4.977 | **Deleterious** | 0.001 | **Damaging** |
| D305H | 1 | **probablydamaging** | -4.972 | **Deleterious** | 0.032 | **Damaging** |
| Y565N | 0.983 | **probablydamaging** | -4.952 | **Deleterious** | 0.127 | **Tolerated** |
| R605G | 0.882 | **possiblydamaging** | -4.94 | **Deleterious** | 0.001 | **Damaging** |
| F311S | 0.236 | **benign** | -4.924 | **Deleterious** | 0.004 | **Damaging** |
| Y443H | 0.999 | **probablydamaging** | -4.767 | **Deleterious** | 0 | **Damaging** |
| T438A | 0.947 | **possiblydamaging** | -4.671 | **Deleterious** | 0.002 | **Damaging** |
| F446V | 0.944 | **possiblydamaging** | -4.667 | **Deleterious** | 0.005 | **Damaging** |
| Y572N | 0.162 | **benign** | -4.667 | **Deleterious** | 0.05 | **Damaging** |
| T438N | 0.887 | **possiblydamaging** | -4.621 | **Deleterious** | 0.001 | **Damaging** |
| M661K | 0.992 | **probablydamaging** | -4.607 | **Deleterious** | 0.001 | **Damaging** |
| G303D | 0.998 | **probablydamaging** | -4.429 | **Deleterious** | 0.003 | **Damaging** |
| G757A | 0.226 | **benign** | -4.407 | **Deleterious** | 0 | **Damaging** |
| Y565C | 0.204 | **benign** | -4.377 | **Deleterious** | 0.091 | **Tolerated** |
| T435I | 0.898 | **possiblydamaging** | -4.319 | **Deleterious** | 0.033 | **Damaging** |
| P352L | 0.559 | **possiblydamaging** | -4.319 | **Deleterious** | 1 | **Tolerated** |
| A441P | 0.967 | **probablydamaging** | -4.156 | **Deleterious** | 0.002 | **Damaging** |
| E527K | 1 | **probablydamaging** | -4 | **Deleterious** | 0 | **Damaging** |
| G868R | 0.735 | **possiblydamaging** | -3.905 | **Deleterious** | 0.005 | **Damaging** |
| R779C | 0.995 | **probablydamaging** | -3.902 | **Deleterious** | 0.012 | **Damaging** |
| K357T | 0.992 | **probablydamaging** | -3.798 | **Deleterious** | 0.002 | **Damaging** |
| T350M | 0.997 | **probablydamaging** | -3.763 | **Deleterious** | 0.055 | **Tolerated** |
| Q798P | 0.045 | **benign** | -3.688 | **Deleterious** | 0.203 | **Tolerated** |
| L634F | 0.998 | **probablydamaging** | -3.667 | **Deleterious** | 0 | **Damaging** |
| T407P | 0.91 | **possiblydamaging** | -3.663 | **Deleterious** | 0.003 | **Damaging** |
| D305N | 1 | **probablydamaging** | -3.624 | **Deleterious** | 0.005 | **Damaging** |
| E569A | 0.077 | **benign** | -3.608 | **Deleterious** | 0.067 | **Tolerated** |
| A441V | 0.97 | **probablydamaging** | -3.594 | **Deleterious** | 0.002 | **Damaging** |
| Q308L | 0.236 | **benign** | -3.504 | **Deleterious** | 0.037 | **Damaging** |
| L355P | 0.975 | **probablydamaging** | -3.502 | **Deleterious** | 0.007 | **Damaging** |
| E306K | 1 | **probablydamaging** | -3.49 | **Deleterious** | 0.044 | **Damaging** |
| D31V | 0.999 | **probablydamaging** | -3.472 | **Deleterious** | 0.007 | **Damaging** |
| G571R | 0.963 | **probablydamaging** | -3.436 | **Deleterious** | 0.036 | **Damaging** |
| R262Q | 0.998 | **probablydamaging** | -3.361 | **Deleterious** | 0.002 | **Damaging** |
| F525L | 0.772 | **possiblydamaging** | -3.333 | **Deleterious** | 0.026 | **Damaging** |
| S333R | 0.051 | **benign** | -3.264 | **Deleterious** | 0.007 | **Damaging** |
| M408V | 0.431 | **benign** | -3.194 | **Deleterious** | 0.044 | **Damaging** |
| E313K | 0.796 | **possiblydamaging** | -3.153 | **Deleterious** | 0.004 | **Damaging** |
| T435A | 0.614 | **possiblydamaging** | -3.144 | **Deleterious** | 0.059 | **Tolerated** |
| M408I | 0.125 | **benign** | -3.113 | **Deleterious** | 0.005 | **Damaging** |
| D90Y | 0.916 | **possiblydamaging** | -3.101 | **Deleterious** | 0.002 | **Damaging** |
| G456E | 0.001 | **benign** | -3.095 | **Deleterious** | 0.072 | **Tolerated** |
| L573F | 0.994 | **probablydamaging** | -3.07 | **Deleterious** | 0.003 | **Damaging** |
| E380K | 0.41 | **benign** | -3.067 | **Deleterious** | 0.045 | **Damaging** |
| K357N | 0.616 | **possiblydamaging** | -3.065 | **Deleterious** | 0.002 | **Damaging** |
| M523T | 0.017 | **benign** | -3.02 | **Deleterious** | 0.001 | **Damaging** |
| I600F | 0.004 | **benign** | -2.979 | **Deleterious** | 0.005 | **Damaging** |
| A354V | 0.989 | **probablydamaging** | -2.942 | **Deleterious** | 0.002 | **Damaging** |
| E640K | 0.402 | **benign** | -2.935 | **Deleterious** | 0.137 | **Tolerated** |
| A441T | 0.454 | **possiblydamaging** | -2.924 | **Deleterious** | 0.05 | **Damaging** |
| S898P | 0 | **benign** | -2.906 | **Deleterious** | 0.027 | **Damaging** |
| P202T | 0.004 | **benign** | -2.902 | **Deleterious** | 0.041 | **Damaging** |
| F581Y | 0.978 | **probablydamaging** | -2.901 | **Deleterious** | 0 | **Damaging** |
| P317L | 0.042 | **benign** | -2.869 | **Deleterious** | 0.011 | **Damaging** |
| R327W | 0.997 | **probablydamaging** | -2.861 | **Deleterious** | 0.004 | **Damaging** |
| D648Y | 0.547 | **possiblydamaging** | -2.839 | **Deleterious** | 0.061 | **Tolerated** |
| Q638E | 0.855 | **possiblydamaging** | -2.794 | **Deleterious** | 0 | **Damaging** |
| F575S | 0.077 | **benign** | -2.79 | **Deleterious** | 0.012 | **Damaging** |
| V264A | 0.004 | **benign** | -2.751 | **Deleterious** | 0.006 | **Damaging** |
| D21H | 1 | **probablydamaging** | -2.725 | **Deleterious** | 0.001 | **Damaging** |
| A497V | 0.281 | **benign** | -2.701 | **Deleterious** | 0.041 | **Damaging** |
| G303S | 1 | **probablydamaging** | -2.596 | **Deleterious** | 0.416 | **Tolerated** |
| S334N | 0.999 | **probablydamaging** | -2.498 | **Neutral** | 0.001 | **Damaging** |
| M70T | 0.996 | **probablydamaging** | -2.487 | **Neutral** | 0.004 | **Damaging** |
| N479K | 0.034 | **benign** | -2.485 | **Neutral** | 0.023 | **Damaging** |
| M664T | 0 | **benign** | -2.438 | **Neutral** | 0.044 | **Damaging** |
| A354T | 0.944 | **possiblydamaging** | -2.422 | **Neutral** | 0.11 | **Tolerated** |
| G781V | 0.857 | **possiblydamaging** | -2.41 | **Neutral** | 0.164 | **Tolerated** |
| R178W | 0.454 | **possiblydamaging** | -2.393 | **Neutral** | 0.024 | **Damaging** |
| R490K | 0.243 | **benign** | -2.355 | **Neutral** | 0.016 | **Damaging** |
| A227V | 0.437 | **benign** | -2.227 | **Neutral** | 0.014 | **Damaging** |
| F525Y | 0.999 | **probablydamaging** | -2.171 | **Neutral** | 0.008 | **Damaging** |
| G309V | 0.89 | **possiblydamaging** | -2.065 | **Neutral** | 0.166 | **Tolerated** |
| I574F | 0.93 | **possiblydamaging** | -2.053 | **Neutral** | 0.091 | **Tolerated** |
| T268P | 0.003 | **benign** | -2.046 | **Neutral** | 0.097 | **Tolerated** |
| F445L | 0.012 | **benign** | -2.036 | **Neutral** | 1 | **Tolerated** |
| K437E | 0.946 | **possiblydamaging** | -2.033 | **Neutral** | 0.007 | **Damaging** |
| G309D | 0.984 | **probablydamaging** | -2.024 | **Neutral** | 0.164 | **Tolerated** |
| L413V | 0.63 | **possiblydamaging** | -2.022 | **Neutral** | 0.005 | **Damaging** |
| A376N | 0.056 | **benign** | -2.021 | **Neutral** | 0.131 | **Tolerated** |
| C150Y | 0 | **benign** | -1.995 | **Neutral** | 0.03 | **Damaging** |
| I521T | 0.03 | **benign** | -1.959 | **Neutral** | 0.482 | **Tolerated** |
| E569K | 0.32 | **benign** | -1.958 | **Neutral** | 0.276 | **Tolerated** |
| I290M | 0.945 | **possiblydamaging** | -1.931 | **Neutral** | 0.031 | **Damaging** |
| F568L | 0.226 | **benign** | -1.931 | **Neutral** | 0.787 | **Tolerated** |
| F311Y | 0.98 | **probablydamaging** | -1.927 | **Neutral** | 0.017 | **Damaging** |
| G571A | 0.249 | **benign** | -1.919 | **Neutral** | 0.062 | **Tolerated** |
| E356D | 0.991 | **probablydamaging** | -1.894 | **Neutral** | 0.011 | **Damaging** |
| E690K | 0.011 | **benign** | -1.876 | **Neutral** | 0.139 | **Tolerated** |
| E629D | 0.589 | **possiblydamaging** | -1.867 | **Neutral** | 0.11 | **Tolerated** |
| K570N | 0.012 | **benign** | -1.853 | **Neutral** | 0.044 | **Damaging** |
| L266F | 0.616 | **possiblydamaging** | -1.726 | **Neutral** | 0.03 | **Damaging** |
| D282N | 0.659 | **possiblydamaging** | -1.718 | **Neutral** | 0.208 | **Tolerated** |
| T195K | 0 | **benign** | -1.651 | **Neutral** | 0.033 | **Damaging** |
| P651S | 0.885 | **possiblydamaging** | -1.649 | **Neutral** | 0.668 | **Tolerated** |
| A77V | 0.304 | **benign** | -1.613 | **Neutral** | 0.034 | **Damaging** |
| D789N | 0.062 | **benign** | -1.583 | **Neutral** | 0.761 | **Tolerated** |
| R170Q | 0.003 | **benign** | -1.571 | **Neutral** | 0.126 | **Tolerated** |
| I174T | 0.027 | **benign** | -1.554 | **Neutral** | 0.004 | **Damaging** |
| K175E | 0.354 | **benign** | -1.546 | **Neutral** | 0.063 | **Tolerated** |
| G811S | 0.006 | **benign** | -1.482 | **Neutral** | 0.506 | **Tolerated** |
| I482F | 0.054 | **benign** | -1.46 | **Neutral** | 0.065 | **Tolerated** |
| L266P | 0.997 | **probablydamaging** | -1.416 | **Neutral** | 0.111 | **Tolerated** |
| L371M | 0.998 | **probablydamaging** | -1.412 | **Neutral** | 0.005 | **Damaging** |
| E692K | 0.145 | **benign** | -1.398 | **Neutral** | 0.727 | **Tolerated** |
| S728G | 0.996 | **probablydamaging** | -1.329 | **Neutral** | 0.138 | **Tolerated** |
| H360R | 0.034 | **benign** | -1.312 | **Neutral** | 1 | **Tolerated** |
| V353L | 0.013 | **benign** | -1.28 | **Neutral** | 0.025 | **Damaging** |
| V353M | 0.249 | **benign** | -1.24 | **Neutral** | 0.008 | **Damaging** |
| Y572F | 0.004 | **benign** | -1.217 | **Neutral** | 0.995 | **Tolerated** |
| G309S | 0.274 | **benign** | -1.208 | **Neutral** | 0.53 | **Tolerated** |
| D213N | 0.02 | **benign** | -1.202 | **Neutral** | 0.069 | **Tolerated** |
| T589I | 0.006 | **benign** | -1.19 | **Neutral** | 0.459 | **Tolerated** |
| Y572H | 0.034 | **benign** | -1.183 | **Neutral** | 0.587 | **Tolerated** |
| R137H | 0.624 | **possiblydamaging** | -1.14 | **Neutral** | 0.1 | **Tolerated** |
| A376D | 0.002 | **benign** | -1.131 | **Neutral** | 0.741 | **Tolerated** |
| M988I | 0.008 | **benign** | -1.059 | **Neutral** | 0.558 | **Tolerated** |
| N913S | 0.001 | **benign** | -1.059 | **Neutral** | 1 | **Tolerated** |
| T195M | 0.001 | **benign** | -1.02 | **Neutral** | 0.167 | **Tolerated** |
| T544M | 0.389 | **benign** | -1.009 | **Neutral** | 0.12 | **Tolerated** |
| M703T | 0 | **benign** | -1.008 | **Neutral** | 0.299 | **Tolerated** |
| S712C | 0.05 | **benign** | -1.004 | **Neutral** | 0.177 | **Tolerated** |
| Q308E | 0.236 | **benign** | -0.936 | **Neutral** | 0.056 | **Tolerated** |
| G328E | 0.994 | **probablydamaging** | -0.923 | **Neutral** | 0.087 | **Tolerated** |
| L266V | 0.786 | **possiblydamaging** | -0.885 | **Neutral** | 0.081 | **Tolerated** |
| I336V | 0.41 | **benign** | -0.799 | **Neutral** | 0.01 | **Damaging** |
| T559A | 0 | **benign** | -0.79 | **Neutral** | 0.277 | **Tolerated** |
| G566S | 0.125 | **benign** | -0.786 | **Neutral** | 0.485 | **Tolerated** |
| R100G | 0 | **benign** | -0.739 | **Neutral** | 0.424 | **Tolerated** |
| Y861C | 0.998 | **probablydamaging** | -0.701 | **Neutral** | 0.075 | **Tolerated** |
| R100H | 0 | **benign** | -0.676 | **Neutral** | 0.154 | **Tolerated** |
| L256M | 0.84 | **possiblydamaging** | -0.653 | **Neutral** | 0.014 | **Damaging** |
| R172S | 0.003 | **benign** | -0.598 | **Neutral** | 0.222 | **Tolerated** |
| L266H | 0.999 | **probablydamaging** | -0.591 | **Neutral** | 0.153 | **Tolerated** |
| Q308K | 0.115 | **benign** | -0.554 | **Neutral** | 1 | **Tolerated** |
| A714S | 0.003 | **benign** | -0.505 | **Neutral** | 0.618 | **Tolerated** |
| R920Q | 0.025 | **benign** | -0.493 | **Neutral** | 0.106 | **Tolerated** |
| Y861H | 0.993 | **probablydamaging** | -0.47 | **Neutral** | 0.183 | **Tolerated** |
| A873T | 0.023 | **benign** | -0.239 | **Neutral** | 0.358 | **Tolerated** |
| S198N | 0.016 | **benign** | -0.156 | **Neutral** | 0.439 | **Tolerated** |
| V200M | 0.037 | **benign** | -0.15 | **Neutral** | 0.162 | **Tolerated** |
| F568Y | 0.994 | **probablydamaging** | -0.099 | **Neutral** | 1 | **Tolerated** |
| Q705K | 0.051 | **benign** | -0.018 | **Neutral** | 0.22 | **Tolerated** |
| H51R | 0.001 | **benign** | 0.052 | **Neutral** | 0.88 | **Tolerated** |
| V72M | 0.989 | **probablydamaging** | 0.082 | **Neutral** | 0.005 | **Damaging** |
| M301V | 0.025 | **benign** | 0.093 | **Neutral** | 0.011 | **Damaging** |
| R327Q | 0.069 | **benign** | 0.169 | **Neutral** | 0.041 | **Damaging** |
| S597G | 0.02 | **benign** | 0.42 | **Neutral** | 0.652 | **Tolerated** |
| L266R | 0.786 | **possiblydamaging** | 0.45 | **Neutral** | 0.915 | **Tolerated** |
| I315V | 0.003 | **benign** | 0.581 | **Neutral** | 0.223 | **Tolerated** |
| H215R | 0.037 | **benign** | 0.684 | **Neutral** | 0.987 | **Tolerated** |
| K377E | 0.002 | **benign** | 0.89 | **Neutral** | 1 | **Tolerated** |
| R550C | 0 | **benign** | 1.037 | **Neutral** | 0.108 | **Tolerated** |
| F304L | 0.091 | **benign** | -4.93 | **Deleterious** | 0.047 | **Damaging** |
| M408T | 0.958 | **probablydamaging** | -5.39 | **Deleterious** | 0.001 | **Damaging** |
| T544I | 0.021 | **benign** | -1.16 | **Neutral** | 0.183 | **Tolerated** |
| K567E | 0.012 | **benign** | -1.1 | **Neutral** | 0.337 | **Tolerated** |
| H314P | 0.002 | **benign** | 1.114 | **Neutral** | 1 | **Tolerated** |
| Prediction metrics: ^a^**PolyPhen-2** Range 0-1; >0.45=possibly damaging; =0.95 probablydamaging. ^b^**PROVEAN**: Range 0-(-N); deleterious =< -2.5. ^c^**SIFT**: Range = 0-1; damaging =< 0.05. (*) | | | | | | |

| **Supplementary Table 2. Ranked Combinatorial Scores for NLRP3 Mutants with p_w_>0.05** | | |
| --- | --- | --- |
| **Site** | **Mutation** | ***p_weighted_*** |
| 357 | K357T | 0.052847965 |
| 605 | R605G | 0.055252239 |
| 303 | G303S | 0.056794415 |
| 438 | T438A | 0.057533576 |
| 525 | F525Y | 0.057699374 |
| 629 | E629G | 0.057764177 |
| 779 | R779C | 0.060226177 |
| 438 | T438N | 0.060948094 |
| 327 | R327W | 0.062280648 |
| 638 | Q638E | 0.062402043 |
| 573 | L573F | 0.062985134 |
| 441 | A441P | 0.063433985 |
| 757 | G757A | 0.064153362 |
| 350 | T350M | 0.069071937 |
| 70 | M70T | 0.069856993 |
| 441 | A441V | 0.070673356 |
| 354 | A354V | 0.072575726 |
| 371 | L371M | 0.076050724 |
| 264 | V264G | 0.077014504 |
| 446 | F446V | 0.077083642 |
| 565 | Y565N | 0.081821882 |
| 607 | E607V | 0.083356709 |
| 407 | T407P | 0.086258704 |
| 355 | L355P | 0.089296562 |
| 90 | D90Y | 0.094785491 |
| 572 | Y572C | 0.107248875 |
| 266 | L266H | 0.109020806 |
| 868 | G868R | 0.114227604 |
| 435 | T435I | 0.114562839 |
| 356 | E356D | 0.115529222 |
| 311 | F311S | 0.118114396 |
| 571 | G571R | 0.118141841 |
| 313 | E313K | 0.119726665 |
| 861 | Y861C | 0.120086316 |
| 266 | L266P | 0.129024129 |
| 357 | K357N | 0.129342172 |
| 72 | V72M | 0.136809893 |
| 311 | F311Y | 0.137280131 |
| 437 | K437E | 0.138346242 |
| 728 | S728G | 0.141881324 |
| 525 | F525L | 0.157200566 |
| 328 | G328E | 0.159729728 |
| 309 | G309D | 0.172145131 |
| 290 | I290M | 0.174502206 |
| 304 | F304L | 0.183905704 |
| 413 | L413V | 0.185300862 |
| 206 | E206G | 0.186301514 |
| 861 | Y861H | 0.190311898 |
| 523 | M523T | 0.192779609 |
| 572 | Y572N | 0.194827256 |
| 354 | A354T | 0.196805249 |
| 574 | I574F | 0.202627101 |
| 435 | T435A | 0.207256076 |
| 408 | M408I | 0.21067505 |
| 568 | F568Y | 0.212738787 |
| 408 | M408V | 0.213241523 |
| 352 | P352L | 0.21956337 |
| 565 | Y565C | 0.22178522 |
| 227 | A227V | 0.226340324 |
| 308 | Q308L | 0.226549748 |
| 178 | R178W | 0.23077461 |
| 333 | S333R | 0.231380585 |
| 309 | G309V | 0.232853704 |
| 256 | L256M | 0.234370103 |
| 380 | E380K | 0.234492367 |
| 441 | A441T | 0.235495662 |
| 781 | G781V | 0.239620175 |
| 648 | D648Y | 0.244464552 |
| 266 | L266F | 0.248857959 |
| 490 | R490K | 0.266831242 |
| 569 | E569A | 0.272215554 |
| 497 | A497V | 0.275728139 |
| 575 | F575S | 0.27634485 |
| 640 | E640K | 0.281783535 |
| 317 | P317L | 0.285266482 |
| 336 | I336V | 0.285315519 |
| 651 | P651S | 0.292260554 |
| 353 | V353M | 0.292770481 |
| 600 | I600F | 0.299803364 |
| 266 | L266V | 0.302745387 |
| 629 | E629D | 0.309445995 |
| 282 | D282N | 0.314531766 |
| 77 | A77V | 0.316979239 |
| 798 | Q798P | 0.317023113 |
| 174 | I174T | 0.320152409 |
| 137 | R137H | 0.321908714 |
| 264 | V264A | 0.328179541 |
| 479 | N479K | 0.336368845 |
| 571 | G571A | 0.348432967 |
| 175 | K175E | 0.348989641 |
| 569 | E569K | 0.382804874 |
| 266 | L266R | 0.40032033 |
| 544 | T544M | 0.400520737 |
| 202 | P202T | 0.403243422 |
| 308 | Q308E | 0.414738507 |
| 898 | S898P | 0.423389695 |
| 376 | A376N | 0.437551313 |
| 456 | G456E | 0.444371577 |
| 482 | I482F | 0.448295178 |
| 353 | V353L | 0.455111291 |
| 301 | M301V | 0.458325547 |
| 568 | F568L | 0.460123982 |
| 309 | G309S | 0.462619682 |
| 327 | R327Q | 0.476408904 |
| 570 | K570N | 0.483995826 |
| 150 | C150Y | 0.510354341 |
| 664 | M664T | 0.513627375 |
| 692 | E692K | 0.515317262 |
| 268 | T268P | 0.536609042 |
| 521 | I521T | 0.536936087 |
| 789 | D789N | 0.537281349 |
| 213 | D213N | 0.539399175 |
| 195 | T195K | 0.558579659 |
| 566 | G566S | 0.559757784 |
| 690 | E690K | 0.560552779 |
| 712 | S712C | 0.564777605 |
| 920 | R920Q | 0.598310958 |
| 544 | T544I | 0.607823189 |
| 200 | V200M | 0.608211169 |
| 705 | Q705K | 0.610417647 |
| 445 | F445L | 0.614813369 |
| 170 | R170Q | 0.617873382 |
| 308 | Q308K | 0.619193849 |
| 572 | Y572H | 0.620012629 |
| 360 | H360R | 0.638993921 |
| 811 | G811S | 0.662790354 |
| 567 | K567E | 0.670713225 |
| 873 | A873T | 0.68205739 |
| 589 | T589I | 0.68786721 |
| 988 | M988I | 0.705104652 |
| 215 | H215R | 0.709457011 |
| 198 | S198N | 0.716096878 |
| 572 | Y572F | 0.736146354 |
| 195 | T195M | 0.742461273 |
| 597 | S597G | 0.744441587 |
| 172 | R172S | 0.760116082 |
| 376 | A376D | 0.780323016 |
| 100 | R100H | 0.799390109 |
| 315 | I315V | 0.803275498 |
| 714 | A714S | 0.812027195 |
| 550 | R550C | 0.814186826 |
| 703 | M703T | 0.819189558 |
| 559 | T559A | 0.833213627 |
| 913 | N913S | 0.844137901 |
| 100 | R100G | 0.853088637 |
| 51 | H51R | 0.892842857 |
| 377 | K377E | 0.915686327 |
| 314 | H314P | 0.920903955 |

| **Supplementary Table 3. FCAS- and CINCA/NOMID-specific Mutants Energies and Notes** | | | | | |
| --- | --- | --- | --- | --- | --- |
| **Variant** | **CINCA/NOMID log(p_w_)** | **FCAS log(p_w_)** | **ΔΔG  (kcal/mol)** | **Evaluation Notes** |  |
| **C261W** |  | -2.4256 | **-1.76** | Opposite side of ATP site |  |
| **L307P** |  | -1.9837 | **-2.37** | Opposite side of ATP site - Pro mutant distorts the helix containing Glu306, which is in salt bridge with Arg262 - causes salt bridge to break and Arg262 to directly interact with PO4 |  |
| **G303D** |  | -1.4733 | **-2.34** | In ATP site - mutation causes shift of Arg262 to directly interact with terminal PO4 of ATP |  |
| M661K |  | -1.4044 |  | Nacht domain - not at protein-protein interface - potential formation of salt bridge with E401 - may alter mobility of NACHT domain |  |
| T438A |  | -1.2401 |  | Opposite end of helix at entrance to ATP site - potential consequence unclear |  |
| E629G |  | -1.2383 |  | Nacht domain hinge region - not at protein-protein interface - disrupts h-bond with Gln624 - may alter mobility of NACHT domain - perhaps making it more floppy / less stable in region around mutation |  |
| Y565N |  | -1.0871 |  | Nacht domain hinge region - not at protein-protein interface - consequence unclear - both residues maintain H-bond with Q638 |  |
| E607V |  | -1.0791 |  | Nacht domain hinge region - not at protein-protein interface but in close proximity - mutation eliminates salt bridge with K617 - perhaps making it more floppy / less stable in region around mutation |  |
| D213N |  | -0.2681 |  | In NBD but on the periphery and not involved in protein-protein interaction - may result in formation of H-bond with D212 or E214 - but functional consequence is unclear |  |
| K377E |  | -0.0383 |  | On periphery of protein but not at protein-protein interaction interface - mutation disrupts salt bridge with E380 - but functional consequences are unclear |  |
| **E527V** | -2.4908 |  | **-2.41** | In ATP site - E527 is in salt bridge with Arg 351 - mutation disrupts this interaction with R351 moves into the active site more and makes contact with the terminal PO4 |  |
| **D305G** | -1.9035 |  | **-1.4** | In ATP site - does not appear to impact ATP binding |  |
| T438P | -1.8794 |  |  | Opposite end of helix at entrance to ATP site - potential consequence unclear - may mildly distort the ATP binding site entrance |  |
| L634F | -1.7069 |  |  | Hinge region of NACHT domain - relatively conservative mutation - could potentially introduce additional pi-pi interactions with Y649 - may decrease flexibility of the hinge |  |
| **E306K** | -1.445 |  | **-2.7** | Already evaluated - enhances ATP binding |  |
| M408T | -1.4378 |  |  | In close proximity to T438 (another identified mutation site) - may introduce an additional H-bond with H522 |  |
| **R262Q** | -1.3793 |  | **-1.49** | Located near ATP binding site - evaluated similar mutation R262W that increased ATP binding affinity - However this mutation did not appear to substantively alter ATP binding |  |
| F525Y | -1.2388 |  |  | F525 is buried next to residue M408 - pretty conservative mutation - functional consequences unclear |  |
| L573F | -1.2008 |  |  | Not at protein-protein interface - in hinge region of Nacht domain - functional consequences are unclear - mutation may introduce pi-pi interactions with F568 |  |
| G757A | -1.1928 |  |  | In LRR domain - functional consequences unclear - addition of methyl R-group may introduce hydrophobic interaction with L748 |  |
| T407P | -1.0642 |  |  | In hinge region of NACHT domain, next to M408 (another identified mutation site) - solvent accessible - mutation may introduce a kink to this helix but functional consequences of this are unclear |  |
| **L266H** | -0.9625 |  |  | At protein-protein interaction interface - may introduce additional H-bond with D321 of neighboring NLRP3 monomer and/or electrostatic interaction depending on charge state of the His mutant |  |
| E356D | -0.9373 |  |  | Not at protein-protein interaction interface - conservative change - functional consequences unclear - maintains H-bond with Q185 |  |
| F311Y | -0.8624 |  |  | Not at protein-protein interaction interface - conservative change - functional consequences unclear - maintains pi-cation interaction with K325 |  |
| S728G | -0.8481 |  |  | Not at protein-protein interaction interface - functional consequences unclear - does not appear to alter interaction pattern in this region |  |
| G328E | -0.7966 |  |  | Located in helix that is part of interaction interface but faces opposite side - points to a mostly hydrophobic internal pocket - doesn't alter interaction pattern - space available to accommodate the mutation so unclear what the functional effect might be |  |
| A354T | -0.706 |  |  | Internal residue not at interaction interface - space available to accommodate mutation - may introduce an extra H-bond with backbone carbonyl of Arg351 |  |
| I574F | -0.6933 |  |  | In hinge domain - conservative change - functional consequences unclear - no change in interaction pattern |  |
| T435A | -0.6835 |  |  | In hinge domain - internal facing residue - does not alter interaction pattern with surrounding residues |  |
| G309V | -0.6329 |  |  | Not located at or near protein-protein interface - located near ATP binding site - functional effects unclear - may introduce additional hydrophobic interactions with Ala310 - unclear what effect that may have on ATP binding |  |
| ***P317L*** | -0.5447 |  |  | Located at start of helix that is part of interaction interface - loss of Pro residue could partially destabilize the helix and impact protein-protein interactions with the neighboring monomer |  |
| V353M | -0.5335 |  |  | Internal residue not at interaction interface - space available to accommodate mutation - conservative change that doesn't alter the interaction pattern in this area |  |
| E629D | -0.5094 |  |  | In hinge domain - conservative change - functional consequences unclear - no change in interaction pattern |  |
| I174T | -0.4946 |  |  | Not at protein-protein interaction interface - functional consequences unclear - mutation loses a hydrophobic interaction with L371 |  |
| V264A | -0.4839 |  |  | Internal residue - somewhat close to interaction interface - relatively conservative change -potential loss hydrophobic interactions with V267 - functional effects unclear |  |
| G571A | -0.4579 |  |  | In hinge domain - conservative change - functional consequences unclear - no change in interaction pattern - space to accommodate the extra methyl group |  |
| ***K175E*** | -0.4572 |  |  | At protein-protein interaction interface - mutation causes loss of salt bridge with D82 on opposing monomer - likely to decrease stability of the oligomer |  |
| **L266R** | -0.3976 |  |  | At protein-protein interaction interface - may introduce additional H-bond with D321 of neighboring NLRP3 monomer and/or electrostatic interaction |  |
| Q308E | -0.3822 |  |  | Located on periphery of ATP site - mutation disrupts one H-bond with Q480 - but it appears unlikely to substantially alter ATP binding - not at protein-protein interaction interface |  |
| A376N | -0.359 |  |  | On periphery of protein - not at interaction interface - mutation does not introduce or disrupt any new interactions |  |
| C150Y | -0.2921 |  |  | On periphery of protein - not at interaction interface - mutation loses an H-bond with the backbone carbonyl of Ser146 but could possibly gain an H-bond between the backbone amine of Ser146 and the phenol moiety of the tyrosine |  |
| M664T | -0.2894 |  |  | In hinge domain - not at interaction interface - on periphery of protein - mutation does not change any interactions |  |
| E690K | -0.2514 |  |  | At interaction interface but despite the radical change the mutation maintains the H-bond with Tyr1012 (although switches from being donated to the E690 to being accepted from the K690 - functional effect likely to be negligible |  |
| F445L | -0.2113 |  |  | Not at protein-protein interaction interface but F445 participates in T-shaped pi-pi interaction with Trp 416 - Trp416 is also not at interaction interface but located on helix that forms part of the interaction interface - L445 eliminates this interaction so could potentially affect the protein-protein interaction |  |
| Q308K | -0.2082 |  |  | Located on periphery of ATP site - somewhat conservative change as mutant can still maintain H-bond with Q480 - appears unlikely to substantially alter ATP binding - not at protein-protein interaction interface |  |
| Y572H | -0.2076 |  |  | Located near ATP binding site - mutation does not alter interaction pattern with surrounding residues - maintains pi interactions with R351 and M523 - functional effect unclear |  |
| T589I | -0.1625 |  |  | In hinge domain - not at or near interaction interface - mutation disrupts an H-bond with Q586 - functional consequence is unclear |  |
| Y572F | -0.133 |  |  | Located near ATP binding site - mutation does not alter interaction pattern with surrounding residues - maintains pi interactions with R351 and M523 - functional effect unclear |  |
| S597G | -0.1282 |  |  | In hinge domain - not at or near interaction interface - mutation disrupts an H-bond with backbone carbonyl of E593 - functional consequence is unclear |  |
| A376D | -0.1077 |  |  | Located at periphery of the protein - not at interaction interface - neither the WT or mutant makes any interactions - functional relevance unclear |  |
| **(Bold)** mutant may alter ATP binding or NLRP3-NLRP3 interaction, ***(Bold italics)*** mutant may ***decrease*** NLRP3-NLRP3 interaction stability | | | | |  |

| **Supplementary Table 4. FATHMM and CADD Bioinformatics Scores of Reported NLRP3 Germline Mutations** | | | | | |
| --- | --- | --- | --- | --- | --- |
| UniProt ID | Variant | FATHMM Score | **FATHMM Predictions^a^** | Raw CADD Score | PHRED CADD Score |
| Q96P20 | T954M | 0.37 | **TOLERATED** | 3.789863 | 26.2 |
| Q96P20 | D21H | 0.27 | **TOLERATED** | 3.494173 | 25 |
| Q96P20 | M70T | 0.38 | **TOLERATED** | 3.475438 | 24.9 |
| Q96P20 | R779C | 0.58 | **TOLERATED** | 3.245269 | 24.2 |
| Q96P20 | E380K | -1.55 | **DAMAGING** | 2.816105 | 23.2 |
| Q96P20 | E607V | -2.38 | **DAMAGING** | 2.719827 | 23 |
| Q96P20 | R327W | -1.46 | **TOLERATED** | 2.510929 | 22.6 |
| Q96P20 | R605G | -2.32 | **DAMAGING** | 2.497281 | 22.6 |
| Q96P20 | M301V | -1.38 | **TOLERATED** | 1.941588 | 19.28 |
| Q96P20 | D282N | -1.25 | **TOLERATED** | 1.818421 | 18.34 |
| Q96P20 | A77V | 0.33 | **TOLERATED** | 1.813483 | 18.3 |
| Q96P20 | M988I | 1.22 | **TOLERATED** | 1.757513 | 17.88 |
| Q96P20 | G811S | 0.54 | **TOLERATED** | 1.733952 | 17.71 |
| Q96P20 | D648Y | -2.35 | **DAMAGING** | 1.704058 | 17.51 |
| Q96P20 | A227V | -1.43 | **TOLERATED** | 1.674243 | 17.31 |
| Q96P20 | V72M | 0.59 | **TOLERATED** | 1.566615 | 16.65 |
| Q96P20 | S728G | 0.6 | **TOLERATED** | 1.420196 | 15.83 |
| Q96P20 | R137H | -0.75 | **TOLERATED** | 1.419563 | 15.82 |
| Q96P20 | R178W | -2.4 | **DAMAGING** | 1.408532 | 15.76 |
| Q96P20 | R327Q | -1.44 | **TOLERATED** | 1.399634 | 15.71 |
| Q96P20 | D789N | 0.68 | **TOLERATED** | 1.333448 | 15.37 |
| Q96P20 | R170Q | -2.28 | **DAMAGING** | 1.065657 | 13.86 |
| Q96P20 | R490K | -3.51 | **DAMAGING** | 0.949383 | 13.08 |
| Q96P20 | P317L | -0.85 | **TOLERATED** | 0.591354 | 10.24 |
| Q96P20 | S898P | 0.57 | **TOLERATED** | 0.577104 | 10.11 |
| Q96P20 | P202T | -2.84 | **DAMAGING** | 0.524658 | 9.632 |
| Q96P20 | I290M | -1.43 | **TOLERATED** | 0.522803 | 9.615 |
| Q96P20 | G456E | -1.67 | **DAMAGING** | 0.436562 | 8.782 |
| Q96P20 | S198N | -2.46 | **DAMAGING** | 0.041643 | 3.285 |
| Q96P20 | H51R | 1.12 | **TOLERATED** | 0.034691 | 3.171 |
| Q96P20 | T559A | -1.75 | **DAMAGING** | 0.011115 | 2.794 |
| Q96P20 | N913S | -0.26 | **TOLERATED** | -0.06056 | 1.816 |
| Q96P20 | A714S | -2.44 | **DAMAGING** | -0.08049 | 1.598 |
| Q96P20 | Q705K | -2.37 | **DAMAGING** | -0.084527 | 1.557 |
| Q96P20 | A873T | 0.81 | **TOLERATED** | -0.096138 | 1.443 |
| Q96P20 | A497V | -2.09 | **DAMAGING** | -0.269068 | 0.452 |
| Q96P20 | M703T | -2.32 | **DAMAGING** | -0.32777 | 0.302 |
| Q96P20 | R550C | -2.35 | **DAMAGING** | -0.371432 | 0.223 |
| Q96P20 | R100G | -0.77 | **TOLERATED** | -0.457371 | 0.121 |
| Q96P20 | T195M | -2.45 | **DAMAGING** | -0.693014 | 0.021 |
| Q96P20 | T195K | -2.43 | **DAMAGING** | -0.708606 | 0.018 |
| Q96P20 | V200M | -2.33 | **DAMAGING** | -0.769166 | 0.011 |
| Q96P20 | I315V | -0.69 | **TOLERATED** | -0.822025 | 0.008 |
| Q96P20 | R100H | -0.78 | **TOLERATED** | -0.862666 | 0.006 |
| Q96P20 | H215R | -1.41 | **TOLERATED** | -1.060385 | 0.002 |
| Q96P20 | T544M | -3.1 | **DAMAGING** | -1.506422 | 0.001 |
| Q96P20 | F525C | -3.99 | **DAMAGING** |  |  |
| Q96P20 | F525Y | -3.88 | **DAMAGING** |  |  |
| Q96P20 | F525L | -3.85 | **DAMAGING** |  |  |
| Q96P20 | G571R | -3.45 | **DAMAGING** |  |  |
| Q96P20 | I521T | -3.43 | **DAMAGING** |  |  |
| Q96P20 | E569A | -3.42 | **DAMAGING** |  |  |
| Q96P20 | E569K | -3.41 | **DAMAGING** |  |  |
| Q96P20 | G571A | -3.4 | **DAMAGING** |  |  |
| Q96P20 | I600F | -3.08 | **DAMAGING** |  |  |
| Q96P20 | L573F | -2.94 | **DAMAGING** |  |  |
| Q96P20 | T544I | -2.9 | **DAMAGING** |  |  |
| Q96P20 | L634F | -2.78 | **DAMAGING** |  |  |
| Q96P20 | S712C | -2.78 | **DAMAGING** |  |  |
| Q96P20 | E527V | -2.64 | **DAMAGING** |  |  |
| Q96P20 | E527K | -2.6 | **DAMAGING** |  |  |
| Q96P20 | E206G | -2.56 | **DAMAGING** |  |  |
| Q96P20 | D305H | -2.44 | **DAMAGING** |  |  |
| Q96P20 | D305G | -2.43 | **DAMAGING** |  |  |
| Q96P20 | D305A | -2.42 | **DAMAGING** |  |  |
| Q96P20 | C150Y | -2.41 | **DAMAGING** |  |  |
| Q96P20 | M661K | -2.41 | **DAMAGING** |  |  |
| Q96P20 | D305N | -2.37 | **DAMAGING** |  |  |
| Q96P20 | T350M | -2.37 | **DAMAGING** |  |  |
| Q96P20 | R172S | -2.34 | **DAMAGING** |  |  |
| Q96P20 | E692K | -2.32 | **DAMAGING** |  |  |
| Q96P20 | M664T | -2.31 | **DAMAGING** |  |  |
| Q96P20 | I174T | -2.27 | **DAMAGING** |  |  |
| Q96P20 | S597G | -2.27 | **DAMAGING** |  |  |
| Q96P20 | E640K | -2.23 | **DAMAGING** |  |  |
| Q96P20 | Y443H | -2.23 | **DAMAGING** |  |  |
| Q96P20 | P651S | -2.2 | **DAMAGING** |  |  |
| Q96P20 | E690K | -2.15 | **DAMAGING** |  |  |
| Q96P20 | K437E | -2.13 | **DAMAGING** |  |  |
| Q96P20 | F446V | -2.07 | **DAMAGING** |  |  |
| Q96P20 | K175E | -2.03 | **DAMAGING** |  |  |
| Q96P20 | W416L | -2 | **DAMAGING** |  |  |
| Q96P20 | T438P | -1.98 | **DAMAGING** |  |  |
| Q96P20 | M408I | -1.96 | **DAMAGING** |  |  |
| Q96P20 | M408T | -1.96 | **DAMAGING** |  |  |
| Q96P20 | T438I | -1.96 | **DAMAGING** |  |  |
| Q96P20 | T438N | -1.94 | **DAMAGING** |  |  |
| Q96P20 | I482F | -1.93 | **DAMAGING** |  |  |
| Q96P20 | M408V | -1.93 | **DAMAGING** |  |  |
| Q96P20 | T438A | -1.89 | **DAMAGING** |  |  |
| Q96P20 | A441P | -1.82 | **DAMAGING** |  |  |
| Q96P20 | A441V | -1.81 | **DAMAGING** |  |  |
| Q96P20 | E629G | -1.81 | **DAMAGING** |  |  |
| Q96P20 | T407P | -1.81 | **DAMAGING** |  |  |
| Q96P20 | F581Y | -1.8 | **DAMAGING** |  |  |
| Q96P20 | T589I | -1.8 | **DAMAGING** |  |  |
| Q96P20 | E629D | -1.79 | **DAMAGING** |  |  |
| Q96P20 | M523T | -1.79 | **DAMAGING** |  |  |
| Q96P20 | Y572C | -1.79 | **DAMAGING** |  |  |
| Q96P20 | A441T | -1.78 | **DAMAGING** |  |  |
| Q96P20 | F568Y | -1.77 | **DAMAGING** |  |  |
| Q96P20 | F575S | -1.77 | **DAMAGING** |  |  |
| Q96P20 | I574F | -1.77 | **DAMAGING** |  |  |
| Q96P20 | Y572F | -1.77 | **DAMAGING** |  |  |
| Q96P20 | G566S | -1.76 | **DAMAGING** |  |  |
| Q96P20 | N479K | -1.76 | **DAMAGING** |  |  |
| Q96P20 | Y565C | -1.76 | **DAMAGING** |  |  |
| Q96P20 | F568L | -1.74 | **DAMAGING** |  |  |
| Q96P20 | T435I | -1.74 | **DAMAGING** |  |  |
| Q96P20 | Y565N | -1.74 | **DAMAGING** |  |  |
| Q96P20 | F445L | -1.73 | **DAMAGING** |  |  |
| Q96P20 | T435A | -1.73 | **DAMAGING** |  |  |
| Q96P20 | Y572N | -1.73 | **DAMAGING** |  |  |
| Q96P20 | Q638E | -1.71 | **DAMAGING** |  |  |
| Q96P20 | L355P | -1.65 | **DAMAGING** |  |  |
| Q96P20 | P352L | -1.64 | **DAMAGING** |  |  |
| Q96P20 | R262W | -1.61 | **DAMAGING** |  |  |
| Q96P20 | L307P | -1.59 | **DAMAGING** |  |  |
| Q96P20 | R262P | -1.59 | **DAMAGING** |  |  |
| Q96P20 | R262L | -1.58 | **DAMAGING** |  |  |
| Q96P20 | R262Q | -1.58 | **DAMAGING** |  |  |
| Q96P20 | K567E | -1.56 | **DAMAGING** |  |  |
| Q96P20 | L413V | -1.56 | **DAMAGING** |  |  |
| Q96P20 | A354V | -1.49 | **TOLERATED** |  |  |
| Q96P20 | E306K | -1.49 | **TOLERATED** |  |  |
| Q96P20 | G303S | -1.49 | **TOLERATED** |  |  |
| Q96P20 | E356D | -1.48 | **TOLERATED** |  |  |
| Q96P20 | L371M | -1.47 | **TOLERATED** |  |  |
| Q96P20 | I336V | -1.46 | **TOLERATED** |  |  |
| Q96P20 | A354T | -1.42 | **TOLERATED** |  |  |
| Q96P20 | A376N | -1.42 | **TOLERATED** |  |  |
| Q96P20 | C261W | -1.34 | **TOLERATED** |  |  |
| Q96P20 | K570N | -1.31 | **TOLERATED** |  |  |
| Q96P20 | F304L | -1.29 | **TOLERATED** |  |  |
| Q96P20 | Y572H | -1.29 | **TOLERATED** |  |  |
| Q96P20 | L256M | -1.27 | **TOLERATED** |  |  |
| Q96P20 | K357T | -1.26 | **TOLERATED** |  |  |
| Q96P20 | V264G | -1.23 | **TOLERATED** |  |  |
| Q96P20 | S333R | -1.21 | **TOLERATED** |  |  |
| Q96P20 | E313K | -1.2 | **TOLERATED** |  |  |
| Q96P20 | V353M | -1.2 | **TOLERATED** |  |  |
| Q96P20 | A376D | -1.19 | **TOLERATED** |  |  |
| Q96P20 | F311Y | -1.19 | **TOLERATED** |  |  |
| Q96P20 | Q308L | -1.19 | **TOLERATED** |  |  |
| Q96P20 | S334N | -1.19 | **TOLERATED** |  |  |
| Q96P20 | V353L | -1.19 | **TOLERATED** |  |  |
| Q96P20 | G309V | -1.18 | **TOLERATED** |  |  |
| Q96P20 | L266V | -1.18 | **TOLERATED** |  |  |
| Q96P20 | T268P | -1.18 | **TOLERATED** |  |  |
| Q96P20 | G328E | -1.17 | **TOLERATED** |  |  |
| Q96P20 | K357N | -1.17 | **TOLERATED** |  |  |
| Q96P20 | L266F | -1.17 | **TOLERATED** |  |  |
| Q96P20 | V264A | -1.16 | **TOLERATED** |  |  |
| Q96P20 | L266H | -1.15 | **TOLERATED** |  |  |
| Q96P20 | L266P | -1.15 | **TOLERATED** |  |  |
| Q96P20 | F311S | -1.14 | **TOLERATED** |  |  |
| Q96P20 | Q308E | -1.11 | **TOLERATED** |  |  |
| Q96P20 | G309D | -1.1 | **TOLERATED** |  |  |
| Q96P20 | G757R | -1.1 | **TOLERATED** |  |  |
| Q96P20 | Q308K | -1.09 | **TOLERATED** |  |  |
| Q96P20 | K377E | -1.04 | **TOLERATED** |  |  |
| Q96P20 | G757A | -1.03 | **TOLERATED** |  |  |
| Q96P20 | G309S | -1.01 | **TOLERATED** |  |  |
| Q96P20 | H360R | -1 | **TOLERATED** |  |  |
| Q96P20 | L266R | -0.94 | **TOLERATED** |  |  |
| Q96P20 | H314P | -0.81 | **TOLERATED** |  |  |
| Q96P20 | D213N | -0.8 | **TOLERATED** |  |  |
| Q96P20 | G303D | -0.78 | **TOLERATED** |  |  |
| Q96P20 | L679P | -0.68 | **TOLERATED** |  |  |
| Q96P20 | R920Q | 0.01 | **TOLERATED** |  |  |
| Q96P20 | D31V | 0.31 | **TOLERATED** |  |  |
| Q96P20 | D90Y | 0.32 | **TOLERATED** |  |  |
| Q96P20 | G868R | 0.36 | **TOLERATED** |  |  |
| Q96P20 | G781V | 0.53 | **TOLERATED** |  |  |
| Q96P20 | Y861C | 0.61 | **TOLERATED** |  |  |
| Q96P20 | Y861H | 0.63 | **TOLERATED** |  |  |
| Q96P20 | Q798P | 0.8 | **TOLERATED** |  |  |
| Prediction metrics: ^a^**FATHMM** Range N-(-N); < -1.5 = damaging; > -1.5 = tolerated. ^b^**CADD**: Range 0-(-N); deleterious =< -2.5. | | | | | |
